# Reversible Linkage of Two Distinct Small Molecule Inhibitors of Myc Generates a Dimeric Inhibitor with Improved Potency That Is Active in Myc Over-Expressing Cancer Cell Lines

**DOI:** 10.1371/journal.pone.0121793

**Published:** 2015-04-15

**Authors:** Jutta Wanner, Darlene Romashko, Douglas S. Werner, Earl W. May, Yue Peng, Ryan Schulz, Kenneth W. Foreman, Suzanne Russo, Lee D. Arnold, Maneesh Pingle, Donald E. Bergstrom, Francis Barany, Stuart Thomson

**Affiliations:** 1 Coferon Inc, 25 Health Sciences Drive, Stony Brook, New York, United States of America; 2 Purdue University, West Lafayette, Indiana, United States of America; 3 Department of Microbiology, Weill Cornell Medical College, New York, New York, United States of America; Lunenfeld-Tanenbaum Research Institute, CANADA

## Abstract

We describe the successful application of a novel approach for generating dimeric Myc inhibitors by modifying and reversibly linking two previously described small molecules. We synthesized two directed libraries of monomers, each comprised of a ligand, a connector, and a bioorthogonal linker element, to identify the optimal dimer configuration required to inhibit Myc. We identified combinations of monomers, termed self-assembling dimeric inhibitors, which displayed synergistic inhibition of Myc-dependent cell growth. We confirmed that these dimeric inhibitors directly bind to Myc blocking its interaction with Max and affect transcription of MYC dependent genes. Control combinations that are unable to form a dimer do not show any synergistic effects in these assays. Collectively, these data validate our new approach to generate more potent and selective inhibitors of Myc by self-assembly from smaller, lower affinity components. This approach provides an opportunity for developing novel therapeutics against Myc and other challenging protein:protein interaction (PPI) target classes.

## Introduction

The use of small molecules as drugs to inhibit cancer targets has made tremendous strides over the last 20 years, with numerous drugs in routine clinical use in a wide range of different cancers. This approach has been particularly successful for enzymatic targets, where the binding site is a well-defined, distinct pocket in the protein that lends itself to the rational design of highly potent inhibitors. However, efforts to expand the use of small molecules to target larger or disordered surface areas that are critical for regulating protein-protein interactions (PPIs) have been met with more limited success. Prototypical PPI inhibitors tend to be large in size and have poor drug like properties and so have limited utility in the clinic. It is clear then that innovative approaches are needed to fully enable the discovery of medicines for the large number of what are generally considered therapeutically relevant but undruggable target classes in many disease areas.

**Fig 1:**
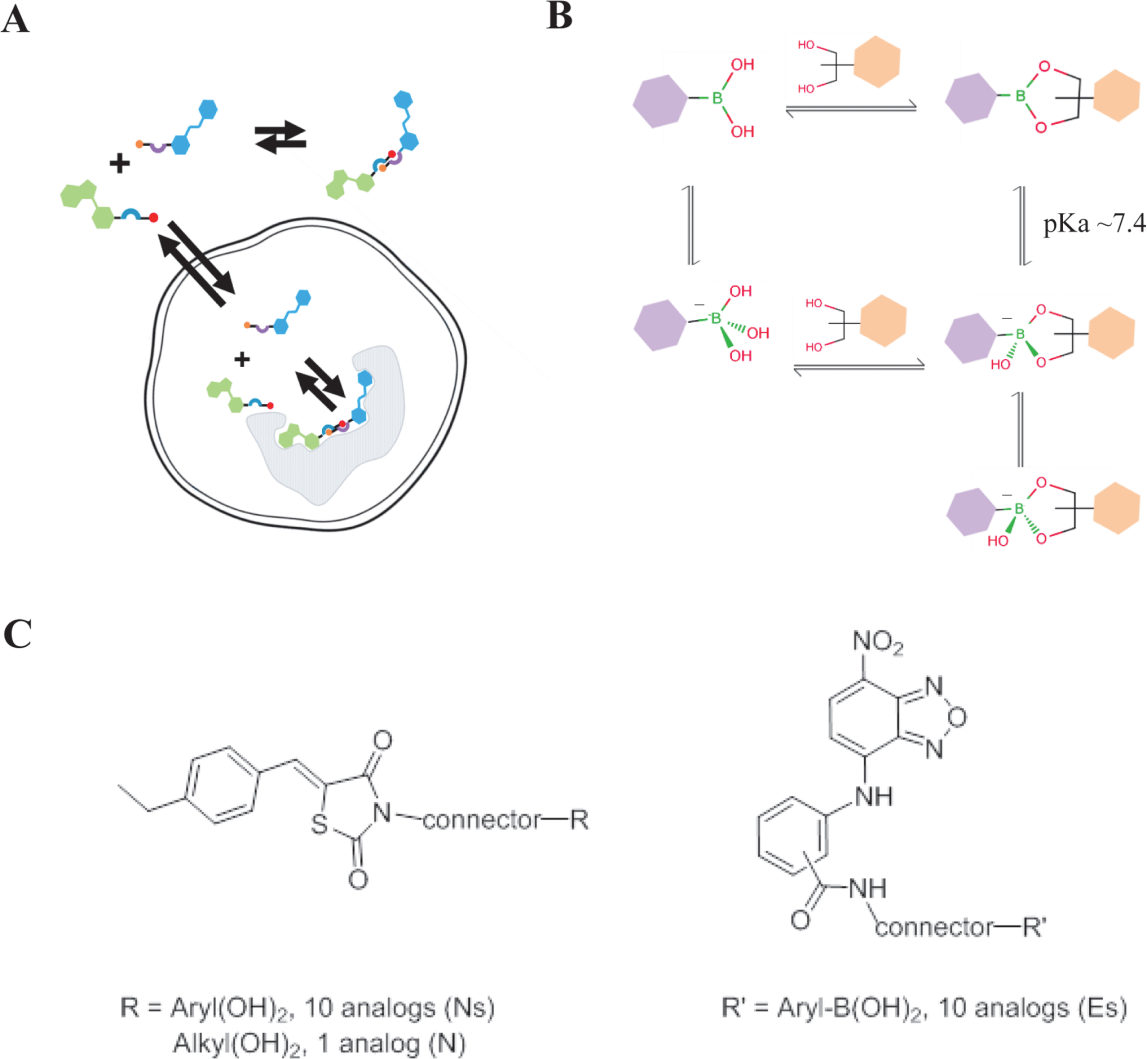
Overview of the basis for generating self-assembling dimeric inhibitors of the Myc transcription factor. A) Schematic representation of the self-assembling dimer approach. Individual monomers (Blue and Green) composed of ligand, connector and a paired bioorthoganol linker are delivered to the cells, cross the plasma membrane and react to form an active dimeric inhibitor in the cells. Dimer assembly may occur in the cellular milieu or on the target of interest. B) Schematic representation of the boronic acid/diol equilibria utilized during formation of dimer. Trigonal planar, neutral species are in equilibrium with the charged chiral tetrahedral species. For a given diol, in the cellular milieu at pH 7.4 the equilibria are determined by the pKas of the boronic acids employed and by the pKas of the boronate esters formed. Racemization of the chiral charged species occurs very rapidly and the biological target will select for the most preferred dimer. C) Summary of library design: Structures of the two parent molecules C01 (left) and C02 (right) and attachment positions; connectors are either alkyl chains or PEG-units; R and R’ are linked to the connectors via amide or carbon bonds; synthetic details of selected library members are provided in the supplementary experimental procedures.

As one approach to address more challenging drug targets we are developing a novel technology to allow self-assembly of small molecules into large dimeric inhibitors, first described by Barany and colleagues [1]. The technology platform enables the delivery of dimeric molecules with a large binding footprint to inhibit biological targets that have frequently challenged traditional medicinal chemistry approaches (Fig. 1A). The dimers are composed of two monomers, each comprising a ligand, a connector, and a bioorthogonal linker element. Under physiological conditions, the monomers may rapidly equilibrate to form dimers through formation of reversible covalent bonds between the linker elements. The linkers are designed to be low molecular weight moieties that can be readily appended to specifically targeted ligands via appropriate connectors. The ligands, linkers and connectors can all be modified to tune the properties of the monomers and allow optimization of good drug-like properties to achieve the desired pharmacokinetic profile. The optimized monomers can be absorbed, distributed to tissues, and enter cells. Once inside the cell, the monomers can bind the target directly, allowing the target to drive self-assembly of the dimer. Alternatively, the monomers can re-equilibrate inside the cell to form the dimer in solution, and the dimer can directly bind and inhibit the target. The extent to which each pathway contributes to the inhibitory effect depends on the intrinsic affinities of the ligands for their respective binding sites on the target, connector length, and the dimerization constant of the linkers employed. Either pathway leads to the target protein being bound by the dimers with a higher affinity and greater specificity than the constituent monomers. The key advantage of this approach is that it allows for the intracellular generation of a large molecule inhibitor, well suited for targeting protein-protein interaction surfaces, while maintaining the ability to capitalize upon the drug-like properties of the small molecule components.

A variety of bioorthogonal linkers are amenable to this technology platform. We and others have described atom-efficient aryl boronic acid linkers that can reversibly dimerize with various catechols and *cis*-alkyl diol partners under aqueous conditions [1, 2, 3, 4] (Fig. 1B). The boronic acids and the partner diols establish equilibrium rapidly, with dimerization constants typically in the μM to mM range [5, 6]. Importantly, the dimerization constant can be adjusted via substituents. For example, the introduction of steric effects on either linker component disfavors boronate ester hydrolysis, shifting the monomer-dimer equilibrium towards dimer formation, which results in improved dimerization constants [7, 8] and can translate into improved potencies of the resulting dimeric inhibitor. Both boronic acid and diol linkers can be appended to desired ligands through a wide range of connector moieties using facile synthetic methods. This technology can be applied to any target comprising two or more proximal binding sites that could be bridged with ligands bearing suitable connectors and linkers. Typically the dimers dissociate from the target with slower off-rates, which leads to prolonged inhibition of the target.

Here we have applied this technology to develop inhibitors against the c-Myc (referred to as Myc hereafter) transcription factor. Myc belongs to a family of transcription factors whose other members include MycL and MycN and these transcription factors have important roles in controlling cell proliferation, survival, and differentiation [9, 10]. Myc is normally tightly regulated but its expression level can be significantly increased in cancer, and this is thought to be a major driver of tumor biology. Myc activity can be deregulated through increased expression by either gene amplification [11] or gene translocation [12]. In more limited cases, particularly in Burkitt’s Lymphoma, the *Myc* gene is mutated [13, 14] which can result in a more stable protein [15, 16]. To function biologically, Myc forms a heterodimer with its partner Max, and the resulting dimer binds to specific promoter motifs, recruits transcription activation complexes, and ultimately activates Myc-dependent genes. It is clear that inactivation of Myc can lead to significant anti-tumor effects in mouse models of cancer [17, 18]. In addition, functional inactivation of Myc in normal tissue using a dominant negative form (OmoMyc) is well tolerated [19], supporting the concept that therapeutically targeting this pathway can be a means to treat cancer.

Numerous direct and indirect methods have been developed to target Myc biology [20]. Recently small molecules that inhibit the BET family of epigenetic reader proteins and impact *Myc* gene expression have shown excellent pre-clinical efficacy in Myc-dependent tumor models [21, 22, 23] and are currently in clinical trials. Several groups have also reported small molecule inhibitors that bind directly to Myc and inhibit its interaction with Max [24, 25]. These inhibitors, originally introduced by Prochownik et al, bind with micromolar affinity and disrupt the Myc:Max interaction, as well as inhibit proliferation of Myc-expressing tumor cell lines. Two such small molecules, 10058-F5 and 10074-G5, have been shown to bind independently and simultaneously to the disordered conformation of the basic helix-loop-helix leucine zipper (bHLHZip) domain of Myc, thus inhibiting its interaction with Max [26, 27, 28]. Additionally, close analogs of 10058-F4 and 10074-G5 with similar and improved potencies have been described [29, 30, 31, 32, 33].

We have utilized our technology platform to develop self-assembling dimeric inhibitors of Myc using these previously described small molecules as our starting individual ligands. These molecules are additionally modified with appended connectors and linkers designed to facilitate reversible dimer formation. We demonstrate that our new inhibitors directly bind to Myc with improved affinity over the existing small molecule inhibitors, disrupt the Myc:Max interaction *in vitro*, and impact expression of MYC regulated genes in cells resulting in anti-proliferative effects in Myc-expressing tumor cell lines.

## Materials and Methods

### Compound Synthesis

A full description of synthetic routes for the molecules described in this paper can be found in S1 File.

### Cell Culture, proliferation and Synergy analysis

K562 (CCL-243), Daudi (CCL-213), Raji (CCL-86) and MV4-11(CCL-9591) cells were purchased directly from American Type Culture Collection (Manassas, VA) and routinely cultured under recommended conditions. Growth and proliferation was determined by use of Cell Titer 96 Aqueous One Solution (Promega, Madison, WI). All cells were plated at 10,000 cells per well in growth media in a clear 96 well plate. After 3 days of compound treatment reagent was added, and absorbance at 490 nm was read after incubation for 4 hours at 37°C. A control plate of compound diluted in media at the same concentrations was treated in a similar way and these values subtracted from the cell plate data to control for any compound interference in the assay. Synergy was determined using the Bliss model of independence.

### Cell lysis and Western blotting

Drug treated cells were washed in PBS and lysed in RIPA buffer (supplemented with protease and phosphatase inhibitors) (Sigma, St. Louis, MO) on ice for 30 minutes. Total protein concentrations were determined using a BCA kit (Thermo Scientific, Rockford, IL). Western blots were performed by resolving proteins by SDS-PAGE and transfer to nitrocellulose membranes. Membranes were probed with antibodies to: c-Myc (9E10) (sc-40); HRP conjugated GAPDH (FL-335) (sc-25778 HRP) (all from Santa Cruz Biotechnology); Max (AF4304) (R&D Systems); Cleaved PARP (Asp214) (D64E10) XP (5625; Cell Signaling). Proteins were visualized using horseradish peroxidase-conjugated secondary Mouse or Rabbit (1:5000) (GE Healthcare) or Goat (1:1000) (R&D Systems) antibodies and the SuperSignal ELISA Femto Maximum Sensitivity Substrate kit was used for detection (Thermo Scientific).

### Surface Plasmon Resonance

All SPR experiments were performed on a Bio-Rad XPR36 instrument at 25°C. His-tagged bHLH-LZ domain of human Myc protein (aa353-439, Cayman Chemicals, Ann Arbor, MI) was immobilized in running buffer (20 mM Na-phosphate, pH 7.5, 300 mM NaCl, 4 mM KCl,. 05% Tween20) in the absence of DMSO at 25μL/min for 400 seconds on Bio-Rad HTE (Ni-NTA) chips with a resulting RU value of about 4500 after immobilization. Compound binding was analyzed in the same running buffer with a final DMSO concentration of 2%. Compounds were injected at 30μL/min for 180 seconds with a dissociation time of 300 seconds. Injections consisted of 5 concentrations of compounds plus a blank channel for reference that flowed over all immobilized ligands in a matrix format. Regeneration steps were not required due to the rapid dissociation of compounds. Equilibrium fits were performed with the ProteOn software after inter-spot and reference channel subtractions.

### Cell-free Myc:Max ELISA

High binding 96-well plates were coated with GST-conjugated full-length Max protein (Sino Biologicals) at 1ng/μL in 100μl of PBS overnight at 4°C. The plate was washed 4x 200 μl/well with PBS and blocked in 200 μl/well of 5% nonfat dry milk in PBS for 2 hours at room temperature. Full length Myc protein (Origene) was diluted to 1 ng/μL in Buffer A (50 mM Tris-HCl, pH 7.4, 0.1 mM EDTA, 150 mM NaCl, 0.002% NP-40). Compounds were serially diluted in 100% DMSO and then sequentially diluted 1:100 into the Myc solution before incubation for one hour at room temperature. For internal consistency, final DMSO concentrations were kept at 2%. The blocked plate was washed 4x 200μl/well with Buffer A, before addition of 100 μl of the Myc compound mixture. Plates were incubated for four hours at RT, and washed 4x 200 μl Buffer A/well. Anti-Myc antibody (Cell Signaling) was diluted 1:1000 in 5% nonfat dry milk in Buffer A. 100 μL/well diluted primary antibody was added to each well and incubated for one hour at room temperature. Plates were washed 4x 200 μl/well Buffer A. HRP-conjugated goat anti-rabbit antibody (GE Healthcare) was diluted 1:5000 in 5% nonfat dry milk in Buffer A. 100 μL/well diluted secondary antibody was added to each well and incubated for thirty minutes at room temperature. The plates were washed 4x 200 μL/well Buffer A. 50 μL/well of FEMTO chemiluminescent reagent (Thermo Scientific) was added, and luminescence was immediately read on a Victor X5 plate reader (PerkinElmer) with a 0.1 sec integration time. IC_50_s were generated through non-linear fitting of data with prism (GraphPad).

### Electrophoretic mobility shift assay (EMSA)

Human c-Myc (NP_002458.2) containing the bHLH-LZ domain was cloned into a modified pET21a vector and then transformed into the BL21StarDE3 expression system, expressed, and purified at Cayman Chemical. The construct consists of an N-terminal 6xHis tag with a TEV cleavage site, c-Myc residues Asn352 to Ala439, and a GGCD C-terminal extension (molecular weight 12.9 kDa). Compounds were added to 200 ng c-Myc in reaction buffer (100 mM KCL, 1 mM DTT, 1 mM EDTA, 50 mM Tris pH7.4, 5mM MgCl_2_, 0.002% NP40) and incubated for 60 minutes at room temperature. 350 ng His-tagged and GST-tagged full-length human Max protein (Sino Biologicals, #12885-H20B) was added, and the reaction was incubated for an additional 60 minutes. Finally, annealed double-stranded E-box containing DNA oligonucleotide with sequence 5’-GATCAGTTGACCACGTGGTCTGGG-3’ was added to a final concentration of 100 nM for twenty minutes incubation at room temperature. The final concentration of DMSO was kept constant at 2%. The protein-DNA complexes (final volume 10 μl) were resolved on NativePAGE Novex 4–16% Bis-Tris Protein Gels (Life technologies, #BN1002BOX) at 4°C with pre-chilled TBE (89 mM Tris-borate, 1 mM EDTA) at 125V for 90 minutes. SYBR Green EMSA Nucleotide Acid gel stain (Molecular Probes, #E33075) was used to stain dsDNA and DNA-protein complexes. Images were captured with an Alpha Innotech FluorChem Q imager installed with AlphaView software.

### RNA Isolation and Real Time-PCR

RNeasy mini kit (Qiagen) was used for RNA purification in accordance with manufacturer’s instructions. First-strand complementary DNA was synthesized and gene expression analyzed using a human Myc-targets PCR array (SABiosciences, #PAHS-177Z) according to the manufacturers protocol.

## Results

### The design and screening of a combinatorial library based on two distinct ligands to identify self-assembling dimeric inhibitors of Myc

We have established a novel technology platform that aims to develop inhibitors versus challenging drug targets through the use of reversible bioorthogonal linker chemistry. Here we have applied this technology to develop inhibitors of the Myc transcription factor. The small molecule inhibitors 10058-F4 and 10074-G5 and their analogs bind to Myc and block its interaction with Max. In addition they have been shown to drive an anti-proliferative effect in Myc driven cell lines at high concentrations [26]. Recently, bivalent probes such as LinkN1 that link the core scaffolds of these molecules were described and are more potent Myc inhibitors compared to either of the individual scaffolds in biochemical and cell assays [30](S1 Fig.). These data suggested that linking these two core scaffolds (referred to here as C01 and C02; S1 Fig.) using our approach could lead to a more potent and selective inhibitor of the Myc transcription factor, with potential for improved monomer pharmacokinetic profiles relative to the larger bivalents. We therefore designed and synthesized two small libraries of monomers by appending selected catechol/alkyl diol and boronic acid linkers via appropriate connectors to the C01 and C02 ligands, respectively (Fig. 1C). We designate monomers bearing boronic acid linkers as “E” (electrophilic) monomers and those bearing catechol or alkyl diol linkers as “N” (nucleophilic) monomers. The libraries contained 10 “E” and 12 “N” monomers respectively, which can interconnect to form 120 dimers, allowing us to efficiently identify “E+N” pairs that most synergistically inhibit Myc. These dimers have maximal spanning distances between their ligands of approximately 7–25Å and feature linker regioisomers for each particular spanning distance.

**Fig 2:**
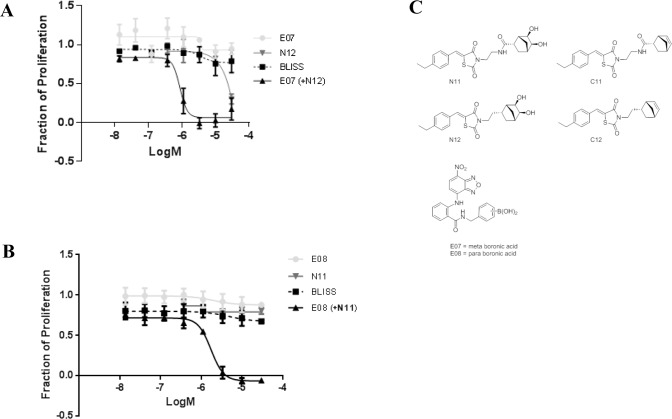
Select combinations of monomers have synergistic activity in a cell proliferation assay. (A and B) Dose-response curves for two different combinations, E07+N12 (A) and E08+N11 (B)tested in the cell proliferation screen. In each case the dose-response curve for each individual monomer is plotted. The dose-response curves for the predicted additive response (Bliss) and the combination experimental data are plotted with an increasing concentration of E07 orE08 in the presence of N11 or N12 (30 μM). The data is plotted as a mean ± SEM from 3 independent experiments.

We screened pairwise combinations of monomers from our libraries in a cell proliferation assay. Increasing concentrations of each compound were dosed in an 8x6 matrix format and their effects on cell proliferation monitored. Our goal was to identify combinations that showed a synergistic inhibition of cell proliferation. The theoretical additive effect on proliferation for each combination, based on the activity of each monomer, was calculated using Bliss analysis [34], and combinations that showed activity greater than this predicted value were considered to be synergistic (S2 Fig.). The majority of the combinations were additive in nature, a result consistent with the combination of the two parent ligands C01 and C02 (S1 Fig.). We identified a number of combinations that were synergistic, and representative graphs showing dose dependent inhibition of proliferation with 2 different combinations (E07+N12 and E08+N11) are shown (Fig. 2 A and B). The structures of these molecules are shown in Fig. 2C. There was no activity of the individual compounds E07, E08, N12, or N11 in the proliferation assay at concentrations up to 30 μM. However, the activity of E07 (Fig. 2A) or E08 (Fig. 2B), was dramatically improved in the presence of 10 μM N12 or 30 μM N11 respectively. In addition, the activity of the E07+N12 and E08+N11 combinations were significantly greater than the predicted additive effect (Bliss line), indicating synergy between these compounds.

These initial screening results indicated that only select combinations were able to drive a synergistic effect in the cell proliferation assay. As the library was initially designed to encompass a range of connector lengths, orientations and dimerization propensities, these results suggest that certain lengths, orientations and linker combinations are preferred to drive the synergistic response in a cellular readout. A more extensive discussion around the structure-activity and structure-property relationships between these different molecules will be presented elsewhere, and so for the purposes of this report we have focused on the combinations that showed the most significant synergy for further validation.

### Myc directed self-assembling dimers bind to Myc and block its interaction with Max

Having identified specific combinations of monomers that were able to drive an anti-proliferative response in Daudi cells, we next confirmed the ability of these monomers to form dimers at the concentration range used in the proliferation assays. We combined the Myc monomeric inhibitors E07 and N11 or E08 and N12 in a 1:1 mixture using 10μM of each compound and analyzed for the presence of dimer using LC-MS. At these concentrations we observed 67% and 24% dimer respectively (S3 and S4 Figs.), confirming that these monomers were able to form significant amounts of dimer at the concentrations used in these experiments.

**Fig 3:**
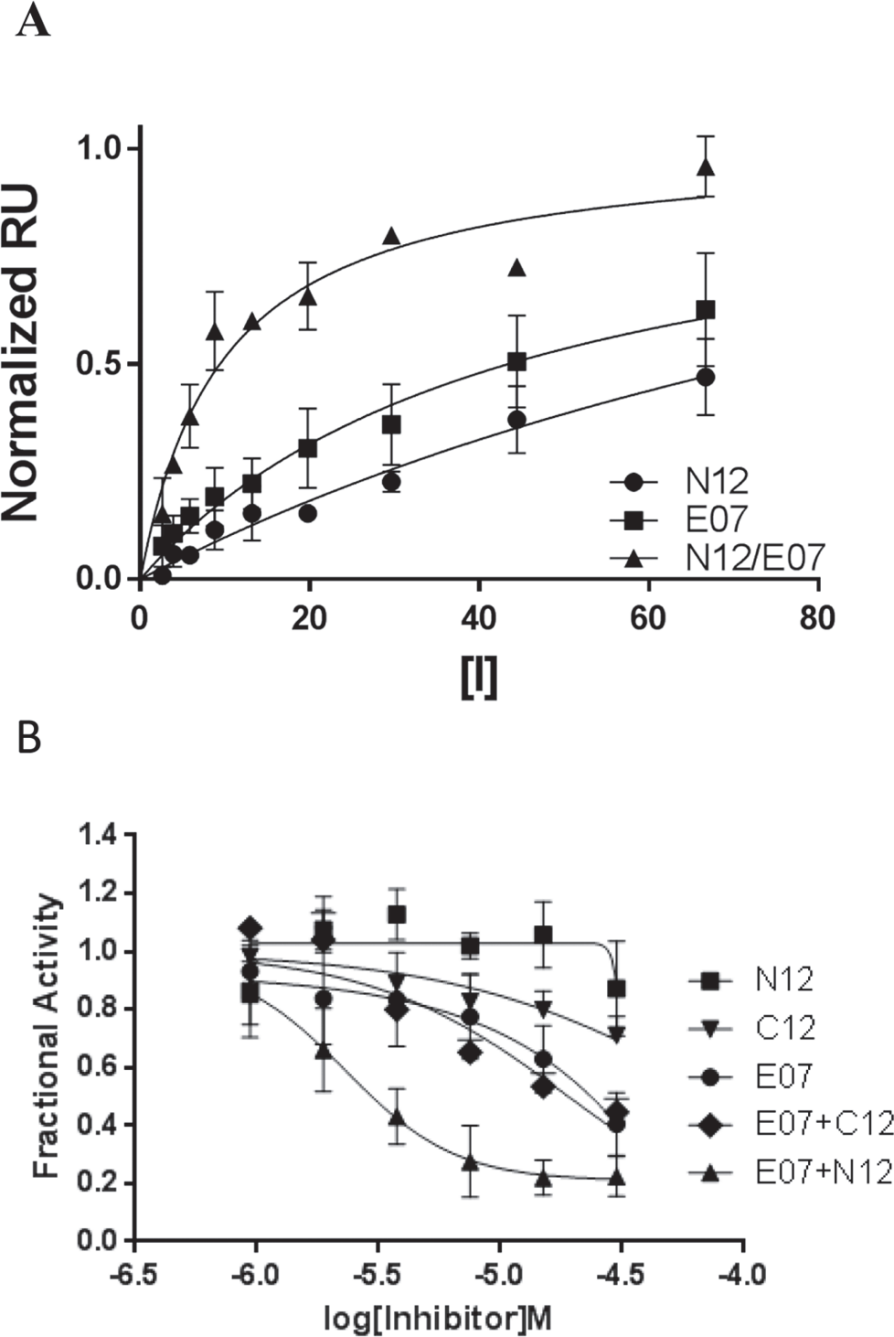
The dimeric inhibitors directly bind to Myc and block its interaction with Max. A) Inhibitors show saturating binding of Myc in SPR experiments. Equilibrium Response Units (RU), normalized to maximal saturated values in individual experiments, are plotted (mean ± SEM) as a function of inhibitor concentration. B) Dose response curves for the inhibition of Myc:Max interaction as determined by ELISA. The data are represented as a fraction of activity compared to a DMSO treated control sample and are plotted as a mean of 2–5 experiments ± SD. The X-axis refers to the concentration of each monomer used.

We next analyzed whether these dimeric inhibitors could directly bind to Myc. To do this we developed a Surface Plasma Resonance (SPR) assay to compare the binding affinities of the monomers to that of the dimeric inhibitors. We immobilized the bHLHZip domain of Myc to the surface of a Ni-NTA chip via a His-tag and measured binding affinities for the monomers or dimers. We observed weak binding of each monomer (mean Kd values E07 = 42 μM and N12 >50 μM. Fig. 3A and Table 1) consistent with a recent report using the parental ligands 10058-F4 and 10078-G5, which showed affinities of 39.7 μM and 31.7 μM respectively for Myc in a similar SPR assay [35]. In contrast, the combination of E07+ N12 bound with a Kd of 8.6 μM, a notable improvement over the individual monomers. We observed similar data with the E08+N11 dimer. Notably, we observed saturation binding of the combinations with stoichiometry of dimer binding less than one, ruling out the possibility that compound aggregation caused by dimerization was responsible for the enhanced binding observed.

**Table 1:** Inhibition of cell-free MYC-MAX heterodimer formation and direct MYC binding*.

	ELISA (IC_50_ )	SPR (K_d_ )
E07	24 ± 7.4	42 ± 10
E08	>30	47 ± 9.0
N11	>30	>50
N12	>30	>50
C11	>30	>50
C12	>30	>50
E07+N12	3.3 ± 1.8	8.6 ± 1.3
E08+N11	12 ± 3.7	17 ± 2.0
E07+C12	>30	>50
E08+C11	>30	>50

*Average IC_50_ values (μM) with standard deviation from the MYC-MAX ELISA and average K_D_ values (μM) from the MYC SPR assay, as described in Experimental Procedures. IC_50_s and K_D_s of E, N, and C monomers alone are listed first, followed by IC_50_s and K_D_s from equimolar titrations of combinations of monomers. C11 and C12 are non-dimerizable control compounds corresponding to N11 and N12, respectively.

In order to confirm that the dimeric inhibitor was driving the improved binding to Myc, we synthesized an analog of N12 that was similar in every aspect except it lacks the diol group required for reaction with its boronic acid counterpart (C12, Fig. 2C). C12 alone or in combination with E07 had Kd values >50 μM (Fig. 3A and Table 1), supporting the conclusion that the ability to form dimer was important for the enhanced binding of these monomers.

We next asked whether the binding of these dimers to Myc could disrupt the interaction with its transcriptional partner Max. We developed an ELISA using purified Myc and Max protein that allowed us to measure effects on Myc:Max binding. The ELISA plate was initially coated with Max protein and the compounds pre-incubated with Myc protein prior to addition to the Max-coated plate. After extensive washing, Myc binding to Max was detected using an anti-Myc antibody. We initially tested the parent ligand molecules, C01 and C02, and observed little inhibition with either of the monomers (IC_50_ >30 μM) or the combination (IC_50_ 23 μM) on the Myc:Max interaction (S1 Table). We next focused on one of our identified dimeric inhibitors, E07+N12 and similarly observed that the individual monomers E07 and N12 showed little inhibition of the Myc:Max interaction (IC_50_ 24 μM and >30 μM respectively; Fig. 3B and Table 1). In contrast, the combination of E07+N12, dosed in a 1:1 ratio, inhibited Myc binding to Max in a dose dependent fashion (IC_50_ 3.3 μM) (Fig. 3B and Table 1), an 8 fold enhancement over the most active individual monomer. We observe similar effects for the dimeric inhibitor E08+N11 (Table 1).

The control combination of C12 with E07 failed to show activity in the Myc:Max ELISA beyond the activity of E07 alone (Fig. 3B), suggesting that the ability of E07+N12 to dimerize was driving the improved inhibitory effect. Limited effects were observed with the additional non-dimerizable control combination E08+C11 (Table 1).

### Self-assembling dimers selectively inhibit Myc:Max binding to DNA

Having confirmed that the dimers bind to Myc and block its binding to Max we next wanted to confirm that these inhibitors selectively blocked the biological activity of Myc in a cell-free assay. The formation of the Myc:Max heterodimer is required for its ability to bind to DNA sequences and trigger transcriptional activation of Myc-dependent genes. Max has the additional capacity to form homodimers that can bind to the same DNA sequences but generally repress gene expression [9, 10]. We therefore performed an electrophoresis gel mobility shift assay to determine if our inhibitors had selectivity for inhibiting the binding of Myc:Max heterodimers to DNA over Max:Max homodimers.

**Fig 4:**
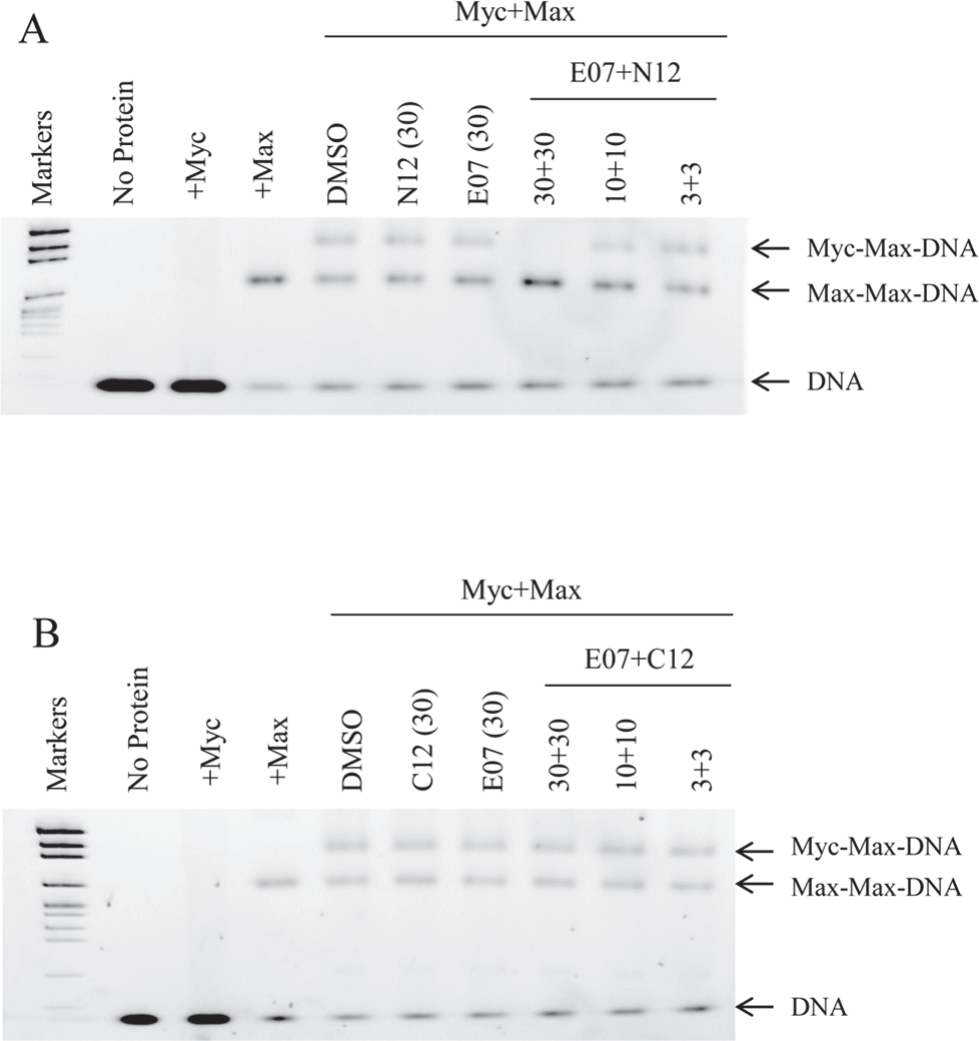
The dimeric inhibitors block Myc:Max but not Max:Max binding to DNA. Gel mobility shift assay showing the effects on Myc:Max DNA complex formation by the dimeric inhibitor E07+N12 (A) and the non-dimerizable control combination E07+C12 (B). The bands that represent protein-DNA complex or naked DNA are shown on the right hand side of each panel. The concentrations indicated are in μM.

Incubation of Max protein with the E-box oligonucleotide resulted in a DNA band shift indicative of Max:Max homodimers (Fig. 4A). Addition of Myc resulted in a decrease in the amount of Max:Max homodimers and the appearance of Myc:Max heterodimers in complex with DNA. Neither of the two monomers, E07 or N12, had any noticeable effect on the Myc:Max or Max:Max complexes, however E07+N12 caused a dose-dependent decrease in the levels of the Myc:Max complex. Notably the decrease in Myc:Max complex is inversely correlated with an increase in the levels of the Max:Max complex, suggesting the dimer is specifically blocking the Myc:Max interaction, freeing Max to homodimerize and bind to the DNA.

As further evidence that the formation of the dimeric inhibitor was critical for the inhibitory activity, we performed similar experiments with the non-dimerizable control monomer C12 (Fig. 4B). In contrast to E07+N12, the combination of E07+C12 had no effect on the binding of the Myc:Max or Max:Max complexes to DNA. These data are consistent with the effects of these compounds in the SPR assay and ELISA.

Collectively these cell-free assay data demonstrate that the self-assembling dimeric inhibitors identified in the cell assays can directly bind to Myc and inhibit its interaction with Max, supporting an on-target mechanism of action for these inhibitors. Further, the use of non-dimerizable control monomers confirms that the ability to form a large molecular weight dimeric inhibitor is key to the ability to target the Myc protein.

### Self-assembly of dimer is critical for cellular activity of Myc inhibitors

**Fig 5:**
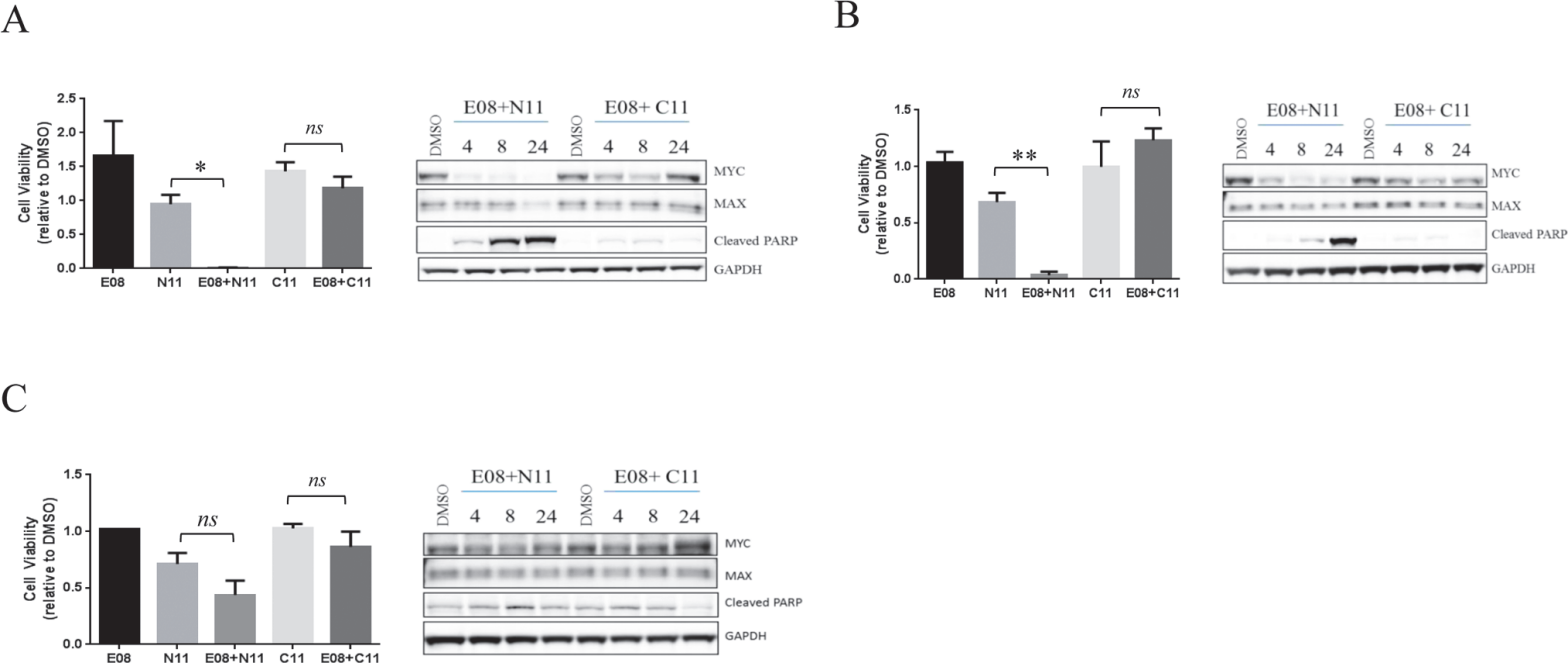
Dimeric inhibitors of Myc drive anti-proliferative effects in Myc over-expressing cell lines that are correlated with a decrease in Myc protein levels. A) Daudi cells were treated with the indicated compounds or combinations for 72 hours and cell viability measured (left panel, * p< 0.05, ** p<0.001, *ns* not significant). In a parallel experiment Daudi cells were treated with E08+N11 or E08+C11 combinations for the indicated times and protein lysates probed with the indicated antibodies (right panel). E08 was used at 10μM and N11 or C11 were used at 30μM. (B) Raji and (C) K562 cells were treated and analyzed as detailed in (A).

Our cell-free experiments had clearly shown that the ability for self-assembly of the dimer was important in driving the improved inhibitory effect versus the Myc protein. To test this in a cellular context we compared the effect on cell viability of the E08+N11 dimer versus its non-dimerizable control combination E08+C11. Treatment of Daudi cells with E08+N11 caused a significant reduction in cell viability after 72 hours of treatment, whereas the E08+C11 combination had no effect on cell viability (Fig. 5A, left panel). Previous reports have indicated that treatment of cells with relatively high concentrations (>50 μM) of the small molecule Myc inhibitors 10058-F4 and 10078-G5 cause a decrease in Myc protein levels [31, 35] and so we used this as a measure of the impact of the dimers on the Myc pathway. Consistent with the effect on cell viability we observed a time-dependent decrease in Myc protein levels, correlated with induction of apoptosis with E08+N11 but not with E08+C11 (Fig. 5A, right panel). Identical results were observed in a second Myc over-expressing cell line Raji (Fig. 5B). In contrast treatment of K562 cells, expressing a BCR-Abl oncogene, with E08+N11 had a modest effect on cell viability, although this was not statistically different from N11 alone, and there was little impact on Myc protein levels (Fig. 5C). Similar effects on cell viability and Myc protein levels were also observed for the dimer E07+N12 but not its non-dimerizable control E07+C12 (S5 Fig.).

To ensure that the decrease in Myc protein levels was selective and not a consequence of extensive protein degradation in the cell we monitored the levels of a number of other proteins, ranging in half life, in the Daudi and Raji cells after treatment with E08+N11 (S6 Fig.). The levels of these proteins are largely unaffected by E08+N11 treatment, other than a consistent modest decrease in protein levels at 24 hours post-treatment in the Daudi cells. This most likely correlates with the early onset of extensive apoptosis in this cell line in contrast to the Raji cells.

### Self-assembling dimers inhibit MYC-dependent gene expression

**Fig 6:**
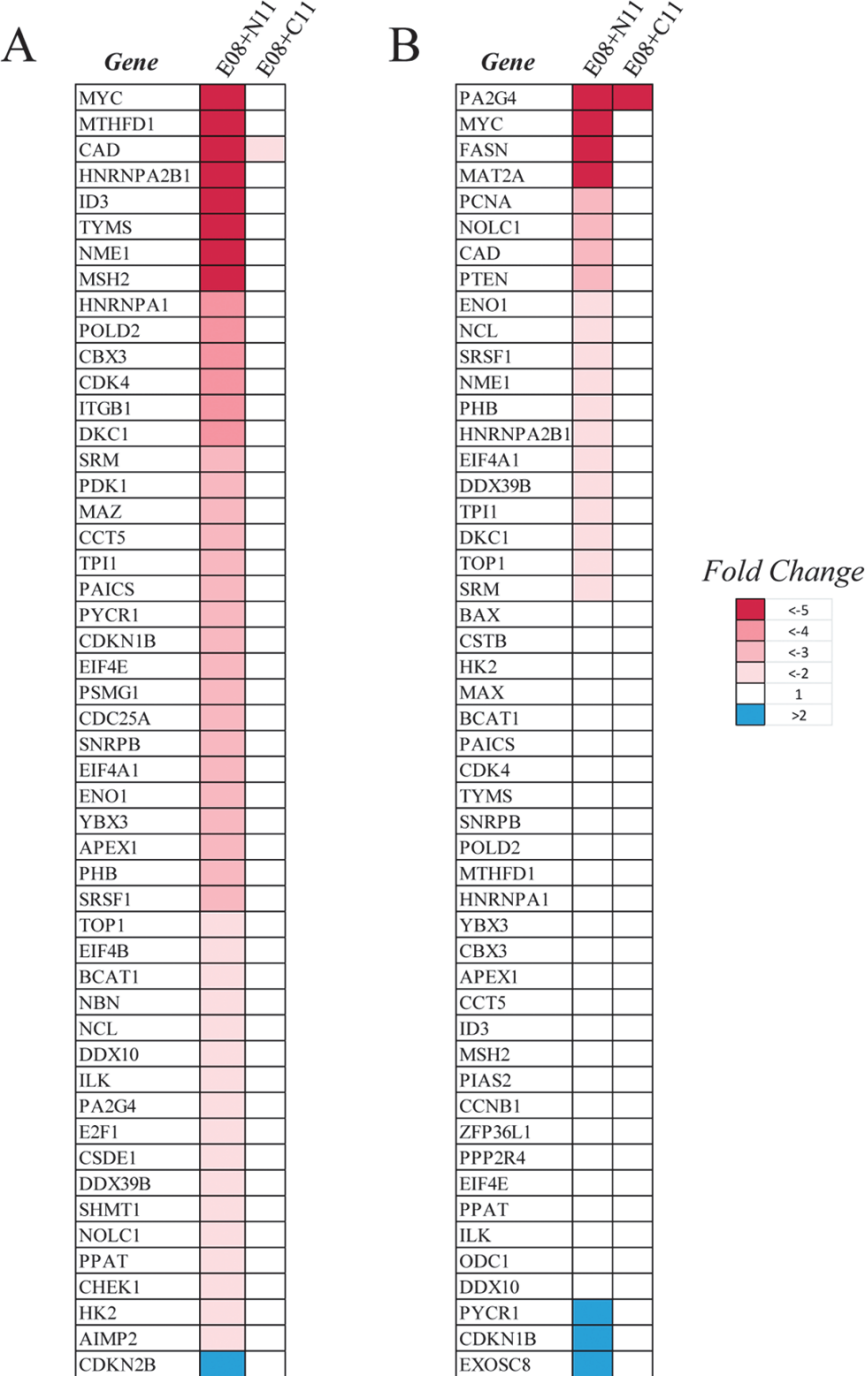
Self-assembling dimeric inhibitor E08+N11, but not the non-dimerizable control E08+C11, inhibits Myc-dependent gene expression. Daudi (A) and Raji (B) cells were treated with E08+N11 or E08+C11 for 24 hours and gene expression levels were analyzed using a human Myc-target PCR array. The 50 genes that change expression the most relative to a DMSO control from each cell line are shown.

Given that the primary role of Myc is to regulate gene expression, we next analyzed the effects of the dimeric inhibitors on a panel of Myc-dependent genes (Fig. 6A). We treated Daudi and Raji cells with the active dimer E08+N11 or its non-dimerizable control E08+C11 and analyzed gene expression using a panel of Myc-dependent genes 24 hours post-treatment. In both cell lines we observed a significant number of genes that were downregulated in response to E08+N11 treatment but not E08+C11, consistent with Myc’s role as a transcriptional activator. A limited number of genes were upregulated in response to treatment but it was notable that in each cell line the cell cycle inhibitors *CDKN2B* or *CDKN1B* genes were upregulated by E08+N11 but not E08+C11, consistent with the effects on cell viability in response to these treatments. Similar effects on Myc-dependent gene expression were observed with the dimeric inhibitor E07+N12 but not its non-dimerizable control E07+C12 (S7 Fig.). Notably the expression of Myc mRNA is decreased in response to E08+N11 in both the Daudi and Raji cells but not in the K562 cells (S7 Fig.), consistent with the effects on Myc steady state protein levels. This suggests that E08+N11 is not acting via a direct inhibition of Myc transcription but that the decrease in Myc mRNA is an indirect consequence of significant inhibition of the Myc function in these cell backgrounds.

## Discussion

Direct targeting of the Myc transcription factor has long been considered a valuable, but largely intractable approach to treating many different types of cancer. Extensive research has been carried out on Myc’s biological function clearly demonstrating its important role in tumor biology, but no approaches have yet resulted in the successful development of therapeutics that directly target Myc. The intrinsic disorder of the bHLHZip domain of monomeric Myc defies structural characterization approaches such as crystallography, and implies the lack of well-defined binding pockets that could be utilized by small molecules to block its biological activity. As such, Myc has long been considered an undruggable target, and recent attention has instead focused on targeting Myc in an indirect manner. In addition to promising RNAi-based approaches (e.g. DCR-M1711), the recent discovery and characterization of small molecule inhibitors (JQ1 and iBET762) which target the BET family of epigenetic reader proteins have proven to be effective in a Myc context [22, 36], and they are currently undergoing evaluation in clinical trials. Due to the pan-BET inhibitory profile of these drugs it is likely they will have wide ranging effects beyond selective Myc inhibition, and so it remains unclear what impact this will have on their clinical safety profile. Thus, a potent and selective inhibitor that directly targets the Myc protein is likely to have significant clinical utility.

We have utilized a novel chemistry platform to identify dimeric inhibitors of Myc. The basis of our approach is to employ bioorthogonal linker chemistries that allow the intracellular self-assembly of two distinct small molecules monomers—each comprising a ligand, a connector and a bioorthogonal linker element—into a large dimeric inhibitor molecule designed to be capable of more potent and selective inhibition of protein:protein interaction targets like Myc. The rapidly reversible nature of the linker chemistry under physiological conditions is such that the small molecule monomeric species are amenable to improvements in their absorption from the gastrointestinal tract, distribution to target tissues, and penetration into the target cell where intracellular dimeric formation can drive more effective Myc inhibition. Since the monomers themselves are optimized for binding to Myc, it is apparent that dimer self-assembly on the target is thermodynamically favored in these circumstances. The key advantage of this approach is that it encompasses the best attributes of small molecules, such as ease of optimization and bioavailability, with the ability to target larger surface areas on the protein of interest, thus enhancing potency and selectivity.

Starting from previously published small molecule inhibitors of Myc that can independently and simultaneously bind to two distinct sites [27, 28], we designed a small directed library to identify dimeric inhibitors of Myc. Our screening approach identified a small number of dimeric inhibitors that bound and inhibited Myc function in biophysical, biochemical, and cellular assays. Importantly, these effects were driven by the ability of successful monomer pairs to dimerize either upon the Myc protein target or through subsequent binding of the preformed dimer to Myc. The small molecules on which we based our library design consistently showed weak activity in our cell-free or cell assays (IC_50_ >30 μM), in agreement with previous reports using similar molecules [26, 29]. Our monomers, with connector and linker groups attached, also show very little activity as individual inhibitors, but the self-assembling dimeric inhibitors have provided a significant improvement in activity.

The dimeric inhibitors appear selective towards Myc for a number of reasons. Firstly, using SPR we demonstrate direct binding of the dimeric inhibitors to Myc with improved affinities in comparison to their constituent monomers. Secondly although the dimerization domains of Myc and Max share extensive structural similarity, our gel-shift experiments demonstrate that the dimeric inhibitors only inhibit the Myc:Max interaction and not the Max:Max interaction, implying selective Myc binding. Of note, our gel shift experiments used the bHLHZip domain of Myc, confirming that our dimers are binding to the same domain first postulated for the original small molecules [27]. Thirdly, only select combinations of monomers from our library were able to demonstrate an inhibition of the Myc:Max interaction, suggesting that connector and linker properties are critical for the formation of an active dimer. It should be noted that this minimizes the possibility that non-specific binding of a large dimer is responsible for the inhibitory effects we observe, as pairs with similar capacity to form a dimer do not show any inhibition of the Myc:Max interaction. Fourthly, we observe anti-proliferative effects with dimers in two Myc over-expressing cell lines but not in a BCR-Abl dependent line. The anti-proliferative effects are correlated with selective decreases in the level of Myc protein, an effect that has previously been observed with 10058-F4 and 10074-G5 like molecules [31, 35]. Finally, we observe an impact on Myc-dependent gene expression with the dimeric inhibitor but not the non-dimerizable control combination, confirming the expected functional consequences of directly targeting the Myc protein. Taken together these data strongly suggest that modifying and reversibly linking these two parents molecules has generated a selective dimeric inhibitor of Myc with enhanced potency over the component monomeric inhibitors.

These first steps in identifying a dimeric inhibitor of Myc provide a good starting point for further optimization to develop an inhibitor that may have the correct attributes to move into clinical testing. As well as modifications in the core ligands to improve target binding properties, as is done with traditional medicinal chemistry approaches, optimization of the connector and linker groups could significantly improve dimerization constants, cell permeability, and the metabolic profile of these inhibitors. Once optimized, there are a large number of indications where these inhibitors may find clinical utility. Many hematological cancers exhibit functional deregulation of Myc through genomic amplification or translocation of the Myc gene while many solid tumors, such as colorectal and lung cancer are also reported to have aberrant function of MYC, primarily caused by genomic amplification [9, 10]. In addition, the Myc family member N-Myc has been shown to be amplified in neuroblastoma and lung cancer, and our dimeric inhibitors would be expected to have activity versus N-Myc as well as Myc due to the high homology between their bHLHZip domains. Indeed, recent reports describe the inhibition of N-Myc in neuroblastoma cell lines with the small molecule 10058-F4 [35, 37], suggesting a similar, more potent effect may also be expected with our dimeric inhibitors.

In summary we have described a novel technology platform that allows for the intracellular generation of large dimeric inhibitors from monomeric components allowing the targeting of challenging or intractable targets inside the cell, exemplified here using Myc as the biological target. This approach is readily adaptable to a wide range of targets, either using pre-existing well-characterized ligands, or newly identified small molecules, that bind to proximal binding sites on their target. We believe that this robust platform can be broadly deployed to deliver potent and highly selective dimeric inhibitors against drug targets that have so far resisted more traditional approaches.

## Supporting Information

S1 File Synthetic routes for compound synthesis. (DOCX)

S1 Fig Activity of parental ligands and bivalents. (A) Structures of C01 (10058-F4 analog), C02 (10074-G5 analog) and the bivalent Link N1 formed by irreversibly linking the two molecules (B) Proliferation assay in Daudi cells showing effects of C01 or C02 alone or the combination of the two molecules dosed in a 1:1 ratio, and LinkN1. The data is presented as a fraction of proliferation with respect to the DMSO treated wells and is a mean ± SEM of two independent experiments each with triplicate wells.
(TIF)

S2 Fig Results of combination screening of monomer libraries. Schematic representation of the results of pairwise combinations of monomers in 72 hour proliferation assay in Daudi cells. Light grey box = no synergy; Dark Grey Box = modest synergy; Black box = significant synergy.
(TIF)

S3 Fig LCMS analysis of monomer-dimer ratios. LCMS (non_polar_3min_1500 run in negative ion mode) profiles of monomers alone or mixtures of monomers in a HEPES pH 7.5 buffer with 2% DMSO. (A) E07 and N12 at 10 μM in a 1:1 ratio (B) N12 at 10 μM (C) E07 at 10μM (D) E07 and C12 at 10 μM in a 1:1 ratio (E) C12 at 10 μM. (F) E07 at 10 μM
(TIF)

S4 Fig LCMS analysis of monomer-dimer ratios. LCMS (non_polar_3min_1500 run in negative ion mode) profiles of monomers alone or mixtures of monomers in a HEPES pH 7.5 buffer with 2% DMSO. (A) E08 and N11 at 10 μM in a 1:1 ratio (B) N11 at 10 μM (C) E08 at 10 μM (D) E08 and C11 at 10 μM in a 1:1 ratio (E) C11 at 10 μM. (F) E08 at 10 μM
(TIF)

S5 Fig Effects of E07+N12 on cell viability and the Myc pathway. (A, C, and E) Daudi (A), MV4-11 (C)) or K562 (E) cells were treated with increasing doses of either E07+N12 or E07+C12 in a 1:1 ratio and the effect on proliferation assayed. The X-axis refers to the concentration of each individual compound, so the total inhibitor concentration will be 2 fold higher at each data point. The data is plotted as a mean ± SEM from 3 independent experiments. (B, D, and F) Daudi (B), MV4-11 (D) or K562 (F) were treated with increasing doses of either E07+N12 or E07+C12 in a 1:1 ratio and the levels of Myc, Max and GAPDH protein analyzed by western blotting after 4 hours of treatment. The relative levels of the Myc protein after correction to GAPDH levels are shown below each Western blot panel.
(TIF)

S6 Fig Effects of Myc dimers on steady state levels of intracellular proteins. Protein lysates from the experiment shown in Fig. 5 were probed with the indicated antibodies. Daudi cells (A) and Raji cells (B) are shown.
(TIF)

S7 Fig Effects of E07+N12 on Myc-dependent gene expression. (A) Daudi cells were treated E07+N12 (10μM + 10 μM) for 4, 8 or 24 hours and the non-dimerizable control combination E07+C12 (10 μM + 10μM) for 24 hours. Gene expression levels were analyzed using a human Myc-target PCR array. Data are representative form two independent experiments. Only those genes that showed expression level changes <-1.8 or >1.8 fold, with respect to DMSO controls, at any time point or with any treatment are shown. (B) Daudi, Raji and K562 cells were treated with E08+N11 or E08+C11 (10 + 30 μM) for 24 hours and the levels of Myc mRNA analyzed by RT-PCR.
(TIF)

S1 Table Parent ligand inhibition of cell-free MYC-MAX heterodimer formation. (DOCX)
