# Supplementary material for: Reversible Linkage of Two Distinct Small Molecule Inhibitors of Myc Generates a Dimeric Inhibitor with Improved Potency That Is Active in Myc Over-Expressing Cancer Cell Lines
Source: PLoS One. 2015 Apr 15;10(4):e0121793. doi: 10.1371/journal.pone.0121793 (PMC4398458; doi:10.1371/journal.pone.0121793)
Supplement: S1 File — (DOCX) [file pone.0121793.s001.docx]

**Supplementary Experimental Procedures**:

Synthesis of compounds C01, C02, C11, C12, E7, E8, N11, and N12:

**General procedures**.  Preparative purification of the compounds were performed on Shimadzu preparative HPLC system composed of the following: CBM-20A system controller, LC-8A binary gradient pump, SPD-M20A photodiode array detector, FRC-10A fraction collector, YMC ODS A 500 X 30 mm X10 μm preparative column using 0.05% (v/v) trifluoroacetic acid in HPLC grade water (A) and 0.05% (v/v) trifluoroacetic acid in HPLC grade acetonitrile (B) at a flow rate of 30.0 mL/min and a run time of 40mins. For basic medium purification, the same instrument was utilized with YMC triart C18, 500X30 mm X 10 μm preparative column using 10 mM ammonium formate and 0.1 % (v/v) ammonia in HPLC grade water (A) and HPLC grade acetonitrile adding 5% (v/v) of mobile phase (A) and 0.1% (v/v) ammonia (B). For both the methods, linear gradient profiles were used depending upon the chromatographic retention and separation of different compounds.

LCMS data was collected on Shimadzu LCMS system equipped with CBM-20A system controller, LC-20AD binary gradient pump, SPD-M20A photodiode array detector, SIL-20AC autosampler, CTO-20AC column oven, LCMS-2010EV single quadrapole mass spectrometer, YMC ODS A 50 X 4.6 mm X 3.0 μm column using 0.05% (v/v) trifluoroacetic acid in HPLC grade water (A) and 0.05% (v/v) trifluoroacetic acid in HPLC grade acetonitrile (B) at a flow rate of 1.2 mL/min and a run time of 5.0 min. The gradient profiles are 20% B to 100% B in 3.0minute, Hold For 0.5 min, at 3.51 min 20% B, Hold until 5.0 min.

HPLC Max plot 210-400 nm.

All Shimadzu LCMS-2010EV instruments utilized electrospray ionization in positive (ES+) or negative (ES-) ionization mode. The Shimadzu LCMS-2010EV instruments can also be utilized with atmospheric pressure chemical ionization in positive (AP+) or negative (AP-) ionization mode.

FINAL COMPOUNDS:

LCMS data for all final compounds were collected on a Waters LCMS system equipped with a Waters Acquity UPLC Sample manager FTN, Waters Acquity UPLC quaternary solvent manager, Waters Acquity UPLC PDA detector and a Waters single quadrapole SQ Detector 2 with ZSpray, ACE Excel 2 SuperC18 100 X 2.1 mm X 2.0 μM column using 0.01% (v/v) formic acid in LCMS grade water (A) and 0.01% (v/v) formic acid in LCMS grade acetonitrile (B) at a flow rate of 0.800 mL/min and a run time of 3 min. Gradient profile (polar_3min_1500): 5% B to 90% B in 1.50 min, hold for 0.5 min, at 2.00 min to 2.20 min 90% B to 5% B, Hold until 3 min. Gradient profile (non_polar_3min_1500): 15% B to 99% B in 1.60 min, hold for 0.6 min, at 2.00 min to 2.80 min 99% B to 15% B, Hold until 3 min.

Waters instrument utilized electrospray ionization in positive (ES+) or negative (ES-) ionization mode

Nuclear Magnetic Resonance (NMR) spectra were recorded on a Varian spectrometer at 400 MHz for proton (^1^H NMR) and 100 MHz for carbon (^13^C NMR); chemical shifts are reported in ppm (δ) relative to residual protons in deuterated solvent peaks.

**Scheme 1: Synthetic scheme for E7, E8 and C02**

**(3-((2-((7-Nitrobenzo[c][1,2,5]oxadiazol-4-yl)amino)benzamido)methyl)phenyl)boronic acid (E7):** A solution of 2-((7-nitrobenzo[c][1,2,5]oxadiazol-4-yl)amino)benzoic acid (**3a**) (150 mg, 0.499 mmol) in DMF (1.5 mL) was charged with EDCI (143 mg, 0.749 mmol), HOBt (101 mg, 0.749 mmol), DIPEA (193 mg, 1.49 mmol) and stirred at room temperature for 10 min. The solution was charged with (3-(aminomethyl) phenyl) boronic acid (**4a**) (83 mg, 0.549 mmol) and stirred at room temperature for 4 h. The reaction mixture was partitioned between DCM (20 mL) and water (10 mL) and separated. The aqueous layer was extracted with DCM (2 X 10 mL) and the combined organic layers were washed with water (2 X 20 mL) and dried over anhydrous Na_2_SO_4_, filtered, and concentrated *in vacuo* resulting in a crude compound which was purified by preparative HPLC purification to afford 32 mg, 15 % yield of the title compound as an orange solid**.** ^1^H NMR (400 MHz, DMSO-d6): δ = 11.74 (s, 1H), 9.30 (br. s., 1H), 8.52 (d, *J* = 8.8 Hz, 1H), 7.98 (s, 2H), 7.89 (d, *J* = 7.5 Hz, 1H), 7.73 – 7.80 (m, 1H), 7.60 – 7.71 (m, 3H), 7.41 (t, *J* = 7.3 Hz, 1H), 7.26 (d, *J* = 7.9 Hz, 1H), 7.13 – 7.19 (m, 1H), 6.88 (d, *J* = 8.8 Hz, 1H), 4.43 (d, *J* = 5.7 Hz, 2H); MS (ES^-^): *m/z* = 431.33, 432.36, 433.36 [M-H]^-^; LCMS: *t*_R_ = 1.89 min (polar_3min_1500).

**(4-((2-((7-Nitrobenzo[c][1,2,5]oxadiazol-4-yl)amino)benzamido)methyl)phenyl)boronic acid (E8):** A solution of 2-((7-nitrobenzo[c][1,2,5]oxadiazol-4-yl)amino)benzoic acid (**3a**) (150 mg, 0.499 mmol) in DMF (1.5 mL) was charged with EDCI (143 mg, 0.749 mmol), HOBt (101 mg, 0.749 mmol), DIPEA (193 mg, 1.49 mmol) and stirred at room temperature for 10 min. The solution was charged with (4-(aminomethyl) phenyl) boronic acid (**4b**) (83 mg, 0.549 mmol) and stirred at room temperature for 4 h. The reaction mixture was partitioned between DCM (20 mL) and water (10 mL) and separated. The aqueous layer was extracted with DCM (2 X 10 mL) and the combined organic layers were washed with water (2 X 20 mL) and dried over anhydrous Na_2_SO_4_, filtered, and concentrated *in vacuo* resulting in a crude compound which was purified by preparative HPLC purification to afford 43 mg, 20 % yield of the title compound as an orange solid**.** ^1^H NMR (400 MHz, DMSO-*d*_6_): δ = 11.71 (s, 1H), 9.29 (t, *J* = 5.5 Hz, 1H), 8.54 (d, *J* = 8.4 Hz, 1H), 7.93 (s, 2H), 7.87 – 7.91 (m, 1H), 7.76 (d, *J* = 7.9 Hz, 1H), 7.63 – 7.70 (m, 3H), 7.41 (t, *J* = 7.5 Hz, 1H), 7.22 (d, *J* = 7.9 Hz, 2H), 6.89 (d, *J* = 8.4 Hz, 1H), 4.44 (d, *J* = 5.7 Hz, 2H); MS (ES^-^): *m/z* = 431.34, 432.33, 433.37 [M-H]^-^; LCMS: *t*_R_ = 1.88 min (polar_3min_1500).

**4-((7-Nitrobenzo[c][1,2,5]oxadiazol-4-yl)amino)benzoic acid (3a):** A solution of 4-chloro-7-nitrobenzo[c][1,2,5]oxadiazole (**1**) (8.0 g, 40.08 mmol) in EtOH (200 mL) was charged with 2-aminobenzoic acid (**2a**) (6.05 mg, 44.09 mmol) and heated at 90°C for 16 h. The reaction mixture was cooled to room temperature upon which a precipitate formed. The precipitate was collected by filtration, washed with diethyl ether followed by pentane and dried *in vacuo* resulting in 4.26 g, 35% yield of the title compound as an orange solid. ^1^H NMR (400 MHz, DMSO-*d*_6_): δ = 12.99 (s, 1H), 11.15 (s, 1H), 8.58 (dd, *J* = 8.7, 2.0 Hz, 1H), 8.04 (dd, *J* = 8.6, 2.1 Hz, 1H), 7.80 - 7.68 (m, 2H), 7.40 (d, *J* = 7.2Hz, 1H), 7.00 (dd, *J* = 8.8, 2.0 Hz, 1H). MS (ES^+^): *m/z* =299.20 [M-H]^+^ ; LCMS: *t*_R_ = 0.47 min.

**2-((7-Nitrobenzo[c][1,2,5]oxadiazol-4-yl)amino)benzamide (C02):**

A solution of 4-chloro-7-nitrobenzo[c][1,2,5]oxadiazole (**1**) (250 mg, 1.25 mmol) in EtOH (15 mL) was charged with 2-aminobenzamide (**2b**) (188 mg, 1.38 mmol) and heated at 90°C for 16 h. The reaction mixture was cooled to room temperature and the precipitate was collected by filtration and washed with diethyl ether followed by pentane and dried *in vacuo* to give 180 mg, 48% yield of the title compound as an orange solid. ^1^H NMR (400 MHz, DMSO-*d*_6_): δ = 12.13 (s, 1H), 8.59 (d, *J* = 8.7 Hz, 1H), 8.33 (s, 1H), 7.92 – 7.76 (m, 3H), 7.66 (td, *J* = 6.8, 1.2 Hz, 1H), 7.35 (td, *J* = 7.2, 1.1 Hz, 1H), 7.04 (d, *J* = 8.8 Hz, 1H); MS (ES^+^): *m/z* =300.00 [M+H]^+^, 322.00 [M+Na]^+^; LCMS: *t*_R_ = 0.48 min.

**Scheme 3: Synthetic scheme for N11**

**(1*R*,2*R*,4*S*,5*S*,6*R*)-*N*-(2-((*Z*)-5-(4-Ethylbenzylidene)-2,4-dioxothiazolidin-3-yl)ethyl)-5,6-dihydroxybicyclo[2.2.1]heptane-2-carboxamide (N11):** A solution of (1*R*,2*R*,4*S*)-*N*-(2-((*Z*)-5-(4-ethylbenzylidene)-2,4-dioxothiazolidin-3-yl)ethyl)bicyclo[2.2.1]hept-5-ene-2-carboxamide (**C11**) (230 mg, 0.580 mmol) in THF:H_2_O (4:1 mL) was charged with NMO (74 mg, 0.632 mmol) and OsO_4_ (22 mg, 0.086 mmol) and stirred at room temperature for 16 h. The reaction mixture was concentrated *in vacuo* resulting in a crude compound which was purified by preparative HPLC to afford 100 mg, 40% yield of the title compound as white solid. ^1^H NMR (400 MHz, DMSO-*d*_6_): δ = 7.97 (t, *J* = 6.0 Hz, 1H), 7.89 (s, 1H), 7.55 (d, *J* = 7.9 Hz, 2H), 7.40 (d, *J* = 8.4 Hz, 2H), 4.54 (br. s., 1H), 4.43 (d, *J* = 4.4 Hz, 1H), 3.62 – 3.79 (m, 2H), 3.60 (br. s., 1H), 3.38 – 3.48 (m, 2H), 3.19 (dd, *J* = 12.8, 6.2 Hz, 1H), 2.67 (q, *J* = 7.5 Hz, 2H), 2.36 – 2.43 (m, 1H), 2.15 (d, *J* = 3.1 Hz, 1H), 1.93 (d, *J* = 4.0 Hz, 1H), 1.71 (d, *J* = 9.3 Hz, 1H), 1.43 (td, *J* = 12.0, 5.1 Hz, 1H), 1.29 – 1.37 (m, 1H), 1.20 (t, *J* = 7.5 Hz, 3H), 1.06 (d, *J* = 9.3 Hz, 1H); MS (ES^-^): *m/z =* 429.38 [M-H]^-^, 475.38 [M+HCOOH]^+^; LCMS: *t*_R_ = 1.98 min (polar_3min_1500).

**(1*R*,2*R*,4*S*)-*N*-(2-((*Z*)-5-(4-Ethylbenzylidene)-2,4-dioxothiazolidin-3-yl)ethyl)bicyclo[2.2.1]hept-5-ene-2-carboxamide (C11):** A solution of (*Z*)-3-(2-aminoethyl)-5-(4-ethylbenzylidene)thiazolidine-2,4-dione (
**10**) (260 mg, 0.942 mmol) in DMF (4 mL) was charged with (1*R*,2*R*,4*S*)-bicyclo[2.2.1]hept-5-ene-2-carboxylic acid (**11**) (129 mg, 0.942 mmol)(Ref: *Science of Synthesis*, vol 6, pg 321-335, **2004**), HATU (536 mg, 1.41 mmol) and DIPEA (364 mg, 2.82mmol) and stirred at room temperature for 2 h. The reaction was diluted with water (10 mL) and extracted with ethyl acetate (3 X 20 mL). The combined organic layers were washed with water (50 mL) and dried over anhydrous Na_2_SO_4_, filtered and concentrated *in vacuo* resulting in a crude compound which was purified by chromatography on silica gel eluting with 2-4% methanol in DCM to give 250 mg, 67% yield of the title compound as white solid. ^1^H NMR (400 MHz, DMSO-*d*_6_): δ = 7.89 (s, 1H), 7.78 (t, *J* = 6.1 Hz, 1H), 7.56 (d, *J* = 8.1 Hz, 2H), 7.40 (d, *J* = 8.1 Hz, 2H), 6.04 (dd, *J* = 5.6, 2.9 Hz, 1H), 5.80 (dd, *J* = 5.4, 2.7 Hz, 1H), 3.67 (t, *J* = 5.4 Hz, 2H), 3.19 – 3.29 (m, 2H), 3.04 (br. s., 1H), 2.78 (br. s., 1H), 2.63 – 2.72 (m, 3H), 1.65 – 1.76 (m, 1H), 1.14 – 1.27 (m, 6H); MS (ES^+^): *m/z =* 397.42, 398.43 [M+H]^+^; LCMS: *t*_R_ = 2.31 min (polar_3min_1500).

**(*Z*)-3-(2-Aminoethyl)-5-(4-ethylbenzylidene)thiazolidine-2,4-dione (10):** A solution of (*Z*)-*tert*-butyl(2-(5-(4-ethylbenzylidene)-2,4-dioxothiazolidin-3-yl)ethyl)carbamate (**9**) (330 mg, 0.876 mmol) in dioxane (3 mL) was cooled to 0°C and charged with 4M HCl in dioxane (5 mL) then stirred at room temperature for 3 h. The reaction mixture was concentrated *in vacuo* resulting in a crude compound which was washed with hexanes and ether and dried *in vacuo* to give 260 mg, 95% yield of title compound as off white solid. ^1^H NMR (400 MHz, DMSO-*d*_6_): δ = 8.07 (s, 2H), 7.91 (s, 1H), 7.57 (d, *J* = 7.9 Hz, 2H), 7.41 (d, *J* = 8.1 Hz, 2H), 3.91 (t, *J* = 6.0 Hz, 2H), 3.07 (d, *J* = 6.3 Hz, 2H), 2.67 (q, *J* = 7.7 Hz, 2H), 1.20 (t, *J* = 7.6 Hz, 3H); MS (ES^+^): *m/z =* 277.10, 278.09, 279.20 [M+H]^+^; LCMS: *t*_R_ = 1.28 min.

**(*Z*)-*tert*-Butyl(2-(5-(4-ethylbenzylidene)-2,4-dioxothiazolidin-3-yl)ethyl)carbamate (9):** A solution of (*Z*)-5-(4-methylbenzylidene)thiazolidine-2,4-dione (**C01**) (650 mg, 2.78 mmol) in acetonitrile (10 mL) was charged with *tert*-butyl (2-bromoethyl)carbamate (**8**) (624 mg, 2.78 mmol) and potassium carbonate (1.15 g, 8.36 mmol) and refluxed at 85 ^o^C for 16 h. The reaction mixture was concentrated *in vacuo* and the residue obtained was diluted with water (20 mL) and extracted with ethyl acetate (3 X 20 mL). The combined organic layers were dried over anhydrous Na_2_SO_4_, filtered and concentrated *in vacuo* resulting in a crude compound which was purified by chromatography on silica gel eluting with 10-30% ethyl acetate in hexanes to give 330 mg, 31% yield of the title compound as off white solid. ^1^H NMR (400 MHz, DMSO-*d*_6_): δ = 7.87 (s, 1H), 7.54 (t, *J* = 9.3 Hz, 2H), 7.40 (d, *J* = 7.7 Hz, 2H), 6.98 (t, *J* = 6.2 Hz, 1H), 3.69 (t, *J* = 5.3 Hz, 2H), 3.18 (q, *J* = 5.8 Hz, 2H), 2.66 (q, *J* = 7.6 Hz, 2H), 1.33 (s, 9H), 1.20 (t, *J* = 7.6 Hz, 3H); MS (ES^+^): *m/z =* 277.14, 278.15 [M-Boc]^+^; LCMS: *t*_R_ = 2.03 min.

**Scheme 2: Synthetic scheme for N12**

**(*Z*)-3-(2-((1*S*,2*S*,4S,5*R*,6*S*)-5,6-Dihydroxybicyclo[2.2.1]heptane-2-yl)ethyl)-5-(4-ethylbenzylidene)thiazolidine-2,4-dione (N12):** A solution of (*Z*)-3-(2-((1*S*,2*S*,4*S*)-bicyclo[2.2.1]hept-5-en-2-yl)ethyl)-5-(4-ethylbenzylidene)thiazolidine-2,4-dione (**C12**) (130 mg, 0.368 mmol) in THF:H_2_O (2:0.5 mL) was charged with NMO (47 mg, 0.405 mmol) and OsO_4_ (14 mg, 0.055 mmol) and stirred at room temperature for 16 h. The reaction was concentrated *in vacuo* resulting in a crude compound which was purified by preparative HPLC to give 55 mg, 38% yield of the title compound as white solid. ^1^H NMR (400 MHz, DMSO-*d*_6_): δ = 7.90 (s, 1H), 7.55 (d, *J* = 7.9 Hz, 2H), 7.39 (d, *J*= 8.4 Hz, 2H), 4.55 (br. s., 2H), 3.79 (d, *J* = 5.6 Hz, 1H), 3.62 (t, *J*= 7.0 Hz, 2H), 3.43 (d, *J* = 5.6 Hz, 1H), 2.66 (q, *J* = 7.6 Hz, 2H), 1.88 – 1.99 (m, 2H), 1.64 – 1.75 (m, 3H), 1.59 (dd, *J* = 13.3, 6.7 Hz, 1H), 1.44 – 1.53 (m, 1H), 1.20 (t, *J* = 7.4 Hz, 3H), 1.00 (d, *J* = 9.8 Hz, 1H), 0.49 (d, *J* = 8.8 Hz, 1H); MS (ES^+^): *m/z =* 388.43 [M+H]^+^, 405.46 [M+H_2_O]; LCMS: *t*_R_ = 2.29 min (polar_3min_1500).

**(*Z*)-3-(2-((1*S*,2*S*,4*S*)-Bicyclo[2.2.1]hept-5-en-2-yl)ethyl)-5-(4-ethylbenzylidene)thiazolidine-2,4-dione (C12):** A solution of (*Z*)-5-(4-methylbenzylidene)thiazolidine-2,4-dione (**C01**) (380 mg, 1.63 mmol) in DMF (20 mL) was cooled to 0^o^C and charged with sodium hydride (61 mg, 2.57 mmol). The reaction mixture was stirred at 0^o^C for 15 min, followed by the addition of 2-((1*R*,2*R*,4*R*)-bicyclo[2.2.1]hept-5-en-2-yl)ethyl 4-methylbenzenesulfonate (**17**) (375 mg, 1.63 mmol) then stirred at room temperature for 14 h. The reaction mixture was diluted with water (10 mL) and extracted with DCM (3 X 20 mL). The combined organic layer was washed with water (30 mL) and dried over anhydrous Na_2_SO_4_, filtered and concentrated *in vacuo* resulting in a crude compound which was purified by chromatography on silica gel eluting with 2-4% ethyl acetate in hexanes to give 130 mg, 28% yield of the title compound as white solid. 1H NMR (400 MHz, DMSO-*d*_6_): δ = 7.90 (s, 1H), 7.55 (d, *J* = 8.1 Hz, 2H), 7.39 (d, *J* = 8.1 Hz, 2H), 6.15 (dd, *J* = 5.6, 2.9 Hz, 1H), 5.89 – 6.00 (m, 1H), 3.63 (t, *J* = 7.3 Hz, 2H), 2.81 (br. s., 1H), 2.74 (br. s., 1H), 2.66 (q, *J* = 7.8 Hz, 2H), 1.92 – 2.02 (m, 1H), 1.80 – 1.90 (m, 1H), 1.40 (dq, *J* = 13.9, 7.2 Hz, 1H), 1.27 – 1.34 (m, 2H), 1.19 (t, *J* = 7.5 Hz, 3H), 0.46 (d, *J* = 10.8 Hz, 1H); MS (ES^+^): *m/z =* no ionization observed; LCMS: *t*_R_ = 2.83 min (nonpolar_3min_1500).

**Scheme 4: Synthetic scheme for compound C01**

**(Z)-5-(4-ethylbenzylidene)thiazolidine-2,4-dione (C01):** A solution of thiazolidine-2,4-dione (**5**) (6.0 g , 51.15 mmol) in ethanol (120 mL) was charged with piperidine (2.18 g, 25.57 mmol) and 4-ethylbenzaldehyde (**6**) (8.24 g, 61.38 mmol) and the solution was heated at 90°C for 16 h. The reaction mixture was cooled to room temperature and evaporated *in vacuo*. The crude product was partitioned between ethyl acetate (100 mL) and water (50 mL) and separated. The aqueous layer was extracted with ethyl acetate (3 x 100 mL) and the combined organic layers were dried over anhydrous Na_2_SO_4_, filtered and concentrated *in vacuo* resulting in a crude compound which was purified by chromatography on silica gel eluting with 10% ethyl acetate in *n-*hexane to obtain 10 g, 84 % yield of the title compound as an off white solid. ^1^H NMR (400 MHz, DMSO-*d*_6_) δ 12.58 (s, 1H, NH), 7.76 (s, 1H, Olefin), 7.52 (d, *J* = 8.2 Hz, 2H), 7.38 (d, *J* = 8.2 Hz, 2H), 2.65 (q, *J* = 7.4 Hz, 2H), 1.19 (t, *J* = 7.4 Hz, 3H). MS (ES+): *m/z* = 232.15 [M-H]^-^; LCMS: *t*_R_ = 2.88 min.

**Scheme 5: Synthetic scheme for intermediate 17**

**2-((1*R*,2*R*,4*R*)-Bicyclo[2.2.1]hept-5-en-2-yl)ethyl 4-methylbenzenesulfonate (17):** A solution of 2-((1*R*,2*R*,4*R*)-bicyclo[2.2.1]hept-5-en-2-yl)ethanol (**16**) (500 mg, 3.62 mmol) in DCM (10 mL) was cooled to 0 ^o^C then charged with triethylamine (424 mg, 4.20 mmol) and tosyl chloride (2.06 g, 10.86 mmol). The reaction mixture was warmed to room temperature and stirred for 16 h. The reaction mixture was concentrated *in vacuo* resulting in a crude compound which was purified by chromatography on silica gel eluting with 2-4% ethyl acetate in hexane to give 700 mg, 66% yield of the title compound as colorless liquid. ^1^H NMR (400 MHz, DMSO-*d*_6_): δ = 7.74 – 7.82 (m, 2H), 7.49 (d, *J* = 7.9 Hz, 2H), 6.10 (dd, *J* = 5.9, 3.0 Hz, 1H), 5.84 (dd, *J* = 5.9, 2.8 Hz, 1H), 3.91 – 4.06 (m, 2H), 2.70 (d, *J* = 3.5 Hz, 1H), 2.62 (s, 1H), 2.45 (s, 3H), 1.97-2.01 (m, 1H), 1.66 – 1.77 (m, 1H), 1.10 – 1.42 (m, 5H).

**2-((1*R*,2*R*,4*R*)-Bicyclo[2.2.1]hept-5-en-2-yl)ethanol (16):** A solution of lithium aluminum hydride (LAH) (271 mg, 7.1 mmol) in THF (10 mL) was cooled 0^o^C and charged with 2-((1*R*,2*R*,4*R*)-bicyclo[2.2.1]hept-5-en-2-yl)acetic acid (**15**) (700 mg, 4.6 mmol) then stirred at room temperature for 2 h. The reaction mixture was quenched with 1N NaOH and ethyl acetate at 0^o^C and filtered through pad of celite. The filtrate was extracted with ethyl acetate (3 X 20 mL) and the combined organic layers were washed with water (30 mL) and dried over anhydrous Na_2_SO_4_, filtered and concentrated *in vacuo* resulting in 500 mg of crude compound as colorless liquid. The crude compound was used in the next step without further purification. ^1^H NMR (400 MHz, DMSO-*d*_6_): δ = 6.12 (dd, *J* = 5.8, 3.0 Hz, 1H), 5.92 (dd, *J* = 5.8, 2.8 Hz, 1H), 4.30 (t, *J* = 5.2 Hz, 1H), 3.32 – 3.41 (m, 2H), 2.69 – 2.71 (m, 2H), 1.94 – 2.11 (m, 1H), 1.72 – 1.87 (m, 1H), 1.02 – 1.38 (m, 5H).

**2-((1*R*,2*R*,4*R*)-Bicyclo[2.2.1]hept-5-en-2-yl)acetic acid (15):** A solution of 2-((1*R*,2*R*,4*R*)-bicyclo[2.2.1]hept-5-en-2-yl)acetonitrile (**14**) (700 mg, 5.26 mmol) in EtOH:H2O (1:1) (6 mL) at room temperature was charged with KOH (884 mg, 15.8 mmol) and refluxed at 95^o^C for 16 h. The solvent was removed *in vacuo* and the residue obtained was acidified with 1N HCl to pH ~2-3 and extracted with DCM (3 X 10 mL). The combined organic layers were dried over anhydrous Na_2_SO_4_, filtered and concentrated *in vacuo* resulting in 700 mg of crude compound as colorless liquid. The crude compound was used in the next step without further purification. ^1^H NMR (400 MHz, DMSO-*d*_6_): δ = 11.94 (s, 1H), 6.17 (dd, *J* = 5.8, 3.0 Hz, 1H), 5.93 (dd, *J* = 5.8, 2.9 Hz, 1H), 2.71 – 2.81 (m, 2H), 2.28 – 2.48 (m, 2H), 2.02 (dd, *J* = 15.4, 7.5 Hz, 1H), 1.70 – 1.95 (m, 2H), 1.16 – 1.38 (m, 2H).

**2-((1*R*,2*R*,4*R*)-Bicyclo[2.2.1]hept-5-en-2-yl)acetonitrile (14):** A solution of (1*S*,2*S*,4*S*)-bicyclo[2.2.1]hept-5-en-2-ylmethyl 4-methylbenzenesulfonate (**13**) (1.5 g, 5.38 mmol) in DMSO (10 mL) at room temperature was charged with sodium cyanide (527 mg, 10.79 mmol) and refluxed at 95 ^o^C for 16 h. The reaction mixture was cooled to room temperature and quenched with water, extracted with ethyl acetate (3 X 50 mL). The combined organic layers were dried over anhydrous Na_2_SO_4_, filtered and concentrated *in vacuo* resulting in 700 mg of crude compound as light yellow liquid. The crude compound was used in the next step without further purification. ^1^H NMR (400 MHz, DMSO-*d*_6_): δ = 6.24 (dt, *J* = 4.6, 2.2 Hz, 1H), 5.97 (dd, *J* = 5.8, 2.9 Hz, 1H), 2.76 – 2.87 (m, 2H), 2.29 – 2.42 (m, 2H), 2.09 – 2.26 (m, 2H), 1.86 – 1.92 (m, 1H), 1.22 – 1.43 (m, 2H).

**(1*S*,2*S*,4*S*)-Bicyclo[2.2.1]hept-5-en-2-ylmethyl 4-methylbenzenesulfonate (13):** A solution of (1*S*,2*S*,4*S*)-bicyclo[2.2.1]hept-5-en-2-ylmethanol (**12**) (760 mg, 6.12 mmol) in DCM (7 mL) was cooled to 0 ^o^C and charged with triethylamine (0.98 mL, 7.11 mmol) and tosyl chloride (4.65 g, 24.5 mmol) then stirred at room temperature for 16 h. The reaction mixture was concentrated *in vacuo* resulting in a crude compound which was purified by chromatography on silica gel eluting with 10-20% ethyl acetate in hexanes to afford 1.5 g, 88% yield of the title compound as colorless liquid.  ^1^H NMR (400 MHz, DMSO-*d*_6_): δ = 7.78 (d, *J* = 8.1 Hz, 2H), 7.49 (d, *J* = 7.9 Hz, 2H), 6.10 (dd, *J* = 5.8, 3.0 Hz, 1H), 5.59 (dd, *J* = 5.8, 2.9 Hz, 1H), 3.74 (dd, *J* = 9.6, 6.5 Hz, 1H), 3.49 (t, *J* = 9.6 Hz, 1H), 2.76 (d, *J* = 13.2 Hz, 2H), 2.31 (s, 3H), 2.28 –2.10 (m, 2H), 1.74 (ddd, *J* = 12.4, 9.3, 3.8 Hz, 1H), 1.26 – 1.34 (m, 1H), 1.20 (d, *J* = 8.2 Hz, 1H).

**(1*S*,2*S*,4*S*)-Bicyclo[2.2.1]hept-5-en-2-ylmethanol (12):** A solution of LAH (533 mg, 14.3 mmol) in THF (10 mL) was cooled to 0^o^C and charged with (1*S*,2*S*,4*S*)-bicyclo[2.2.1]hept-5-en-2-carboxylic acid (**11**) (1.25 g, 9.05 mmol). The reaction was stirred at room temperature for 2 h then cooled to 0 ^o^C and quenched with 1N NaOH and ethyl acetate. The reaction mixture was filtered through pad of celite and the filtrate was extracted with ethyl acetate (3 X 50 mL). The combined organic layers were washed with water (50 mL) and dried over anhydrous Na_2_SO_4_, filtered and concentrated *in vacuo* resulting in 760 mg of crude compound as colorless liquid. The crude compound was used in the next step without further purification. ^1^H NMR (400 MHz, DMSO-*d*_6_): δ = 6.10 (dd, *J* = 5.5, 2.9 Hz, 1H), 5.92 (dd, *J* = 6.0, 2.9 Hz, 1H), 4.36 (t, *J* = 5.3 Hz, 1H), 3.11 (dt, *J* = 11.1, 5.8 Hz, 1H), 2.93 (td, *J* = 9.7, 5.3 Hz, 1H), 2.84 (s, 2H), 2.73 (d, *J* = 5.3 Hz, 1H), 2.16 (tt, *J* = 9.1, 4.4 Hz, 1H), 1.71 (ddd, *J* = 12.5, 9.1, 3.8 Hz, 1H), 1.16 – 1.38 (m, 2H).
