# Supplementary figures and images for: Reversible Linkage of Two Distinct Small Molecule Inhibitors of Myc Generates a Dimeric Inhibitor with Improved Potency That Is Active in Myc Over-Expressing Cancer Cell Lines
Source: PLoS One. 2015 Apr 15;10(4):e0121793. doi: 10.1371/journal.pone.0121793 (PMC4398458; doi:10.1371/journal.pone.0121793)

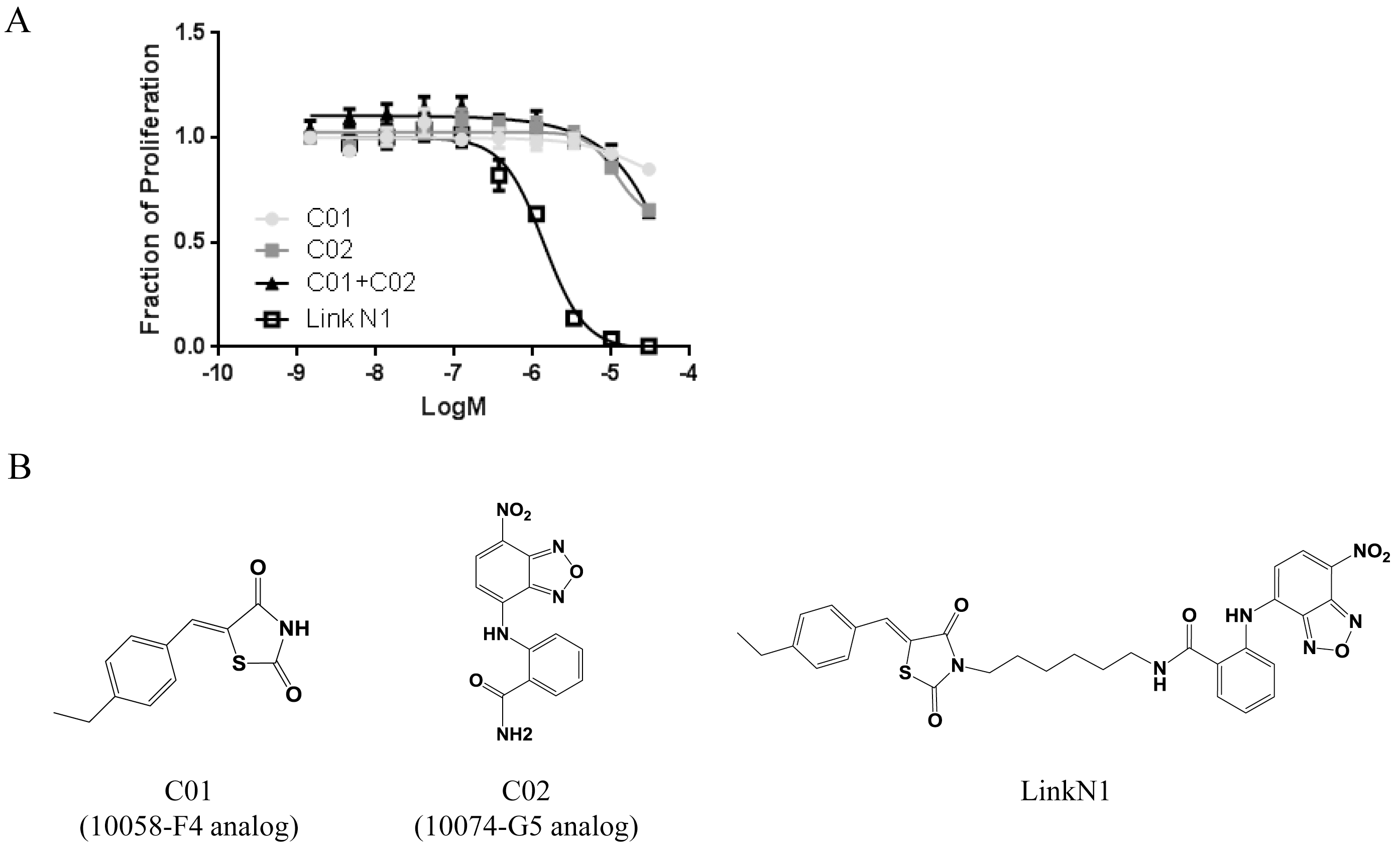

Supplement: S1 Fig — (A) Structures of C01 (10058-F4 analog), C02 (10074-G5 analog) and the bivalent Link N1 formed by irreversibly linking the two molecules (B) Proliferation assay in Daudi cells showing effects of C01 or C02 alone or the combination of the two molecules dosed in a 1:1 ratio, and LinkN1. The data is presented as a fraction of proliferation with respect to the DMSO treated wells and is a mean ± SEM of two independent experiments each with triplicate wells. (TIF) [file pone.0121793.s002.tif]

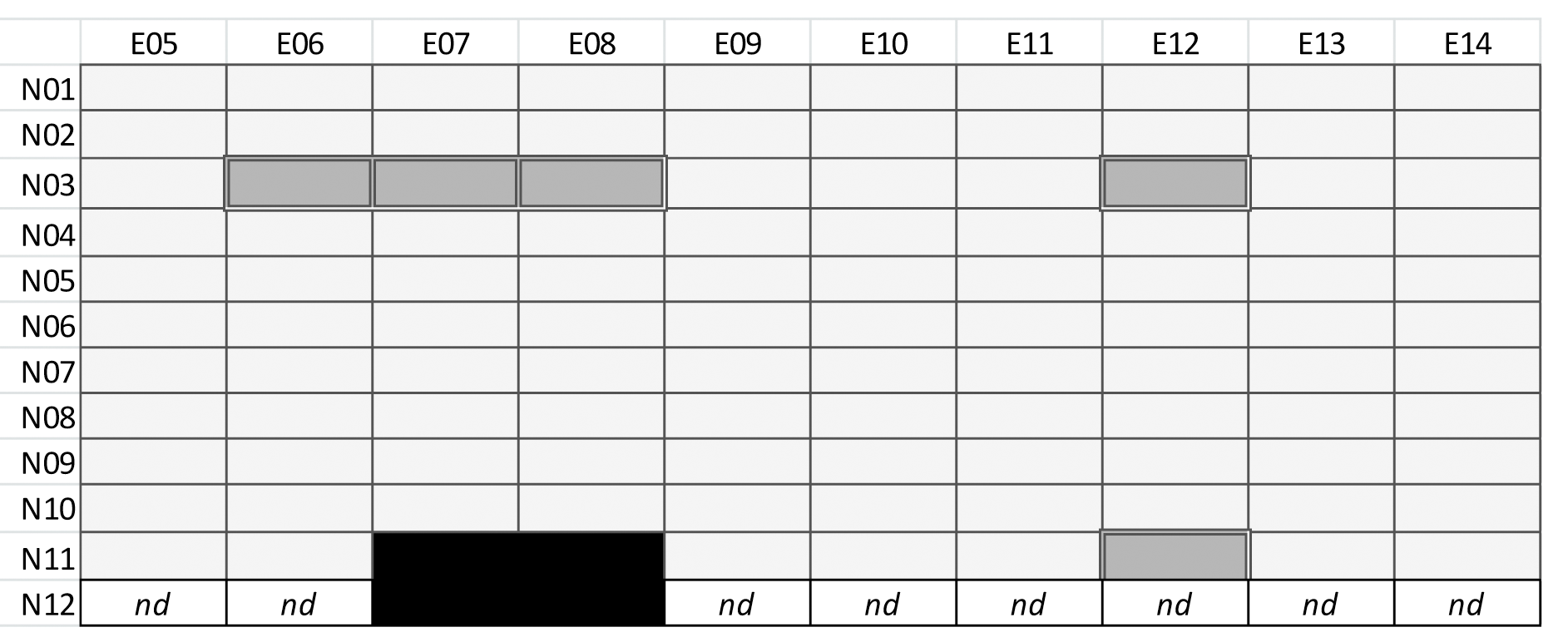

Supplement: S2 Fig — Schematic representation of the results of pairwise combinations of monomers in 72 hour proliferation assay in Daudi cells. Light grey box = no synergy; Dark Grey Box = modest synergy; Black box = significant synergy. (TIF) [file pone.0121793.s003.tif]

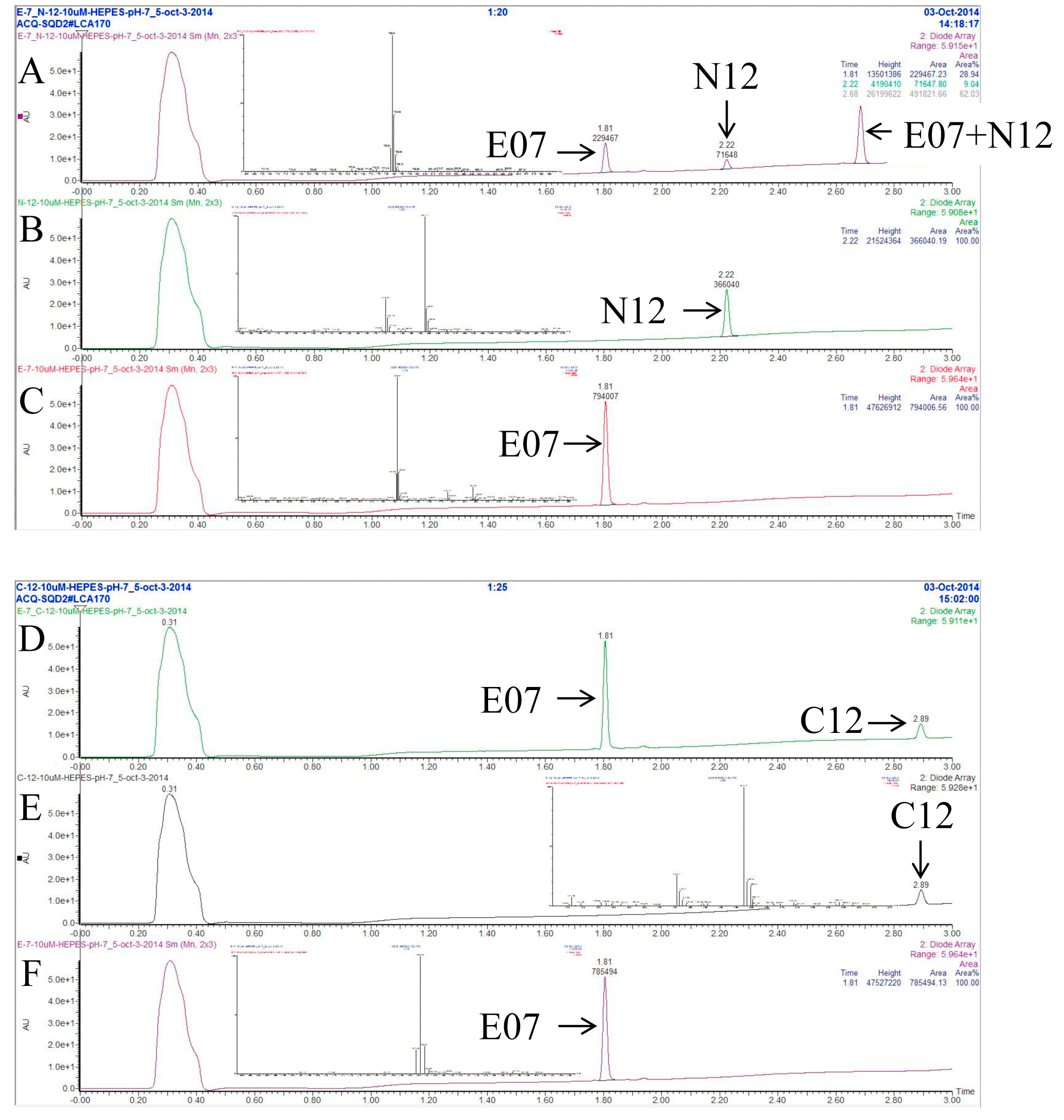

Supplement: S3 Fig — LCMS (non_polar_3min_1500 run in negative ion mode) profiles of monomers alone or mixtures of monomers in a HEPES pH 7.5 buffer with 2% DMSO. (A) E07 and N12 at 10 μM in a 1:1 ratio (B) N12 at 10 μM (C) E07 at 10μM (D) E07 and C12 at 10 μM in a 1:1 ratio (E) C12 at 10 μM. (F) E07 at 10 μM (TIF) [file pone.0121793.s004.tif]

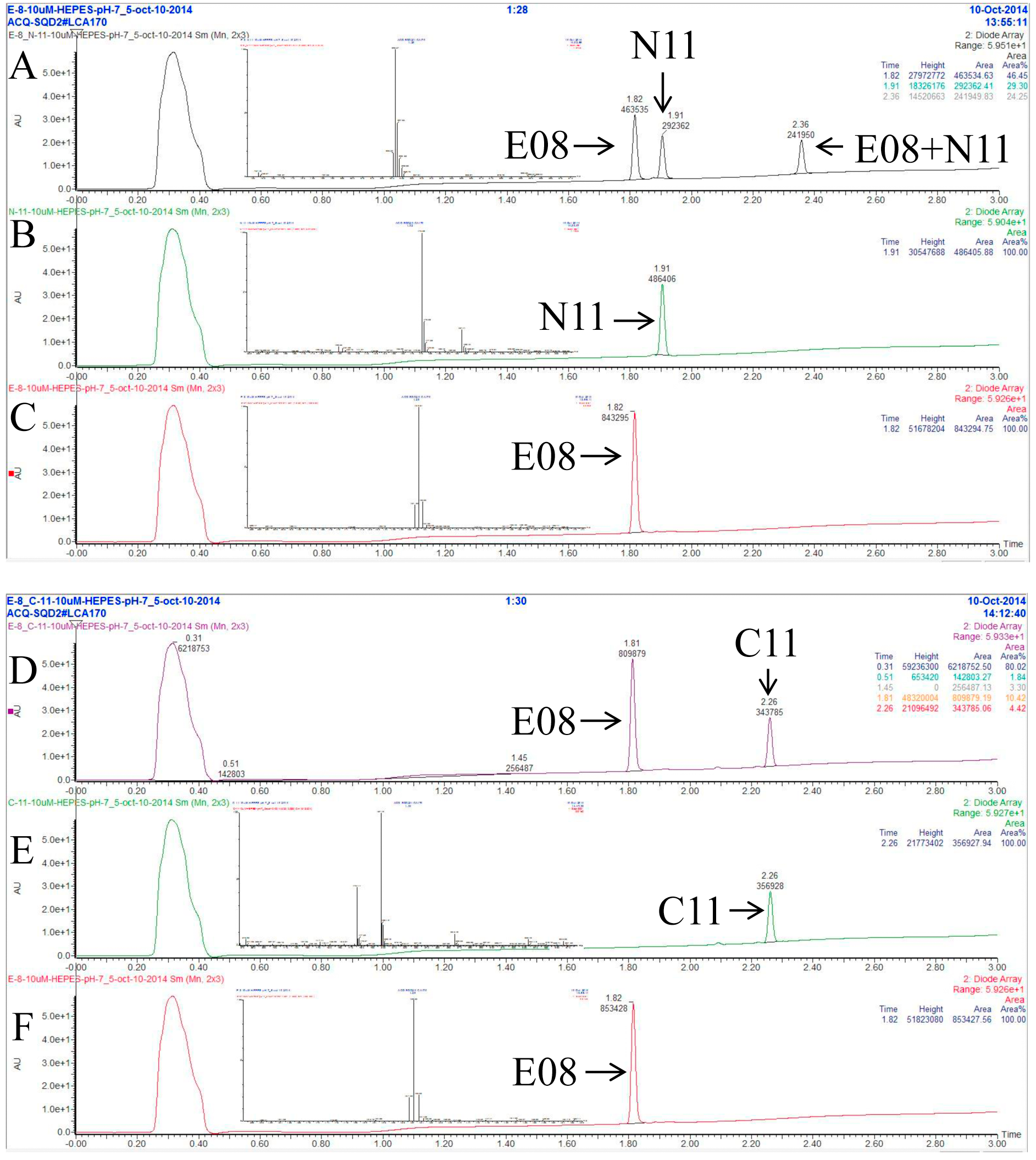

Supplement: S4 Fig — LCMS (non_polar_3min_1500 run in negative ion mode) profiles of monomers alone or mixtures of monomers in a HEPES pH 7.5 buffer with 2% DMSO. (A) E08 and N11 at 10 μM in a 1:1 ratio (B) N11 at 10 μM (C) E08 at 10 μM (D) E08 and C11 at 10 μM in a 1:1 ratio (E) C11 at 10 μM. (F) E08 at 10 μM (TIF) [file pone.0121793.s005.tif]

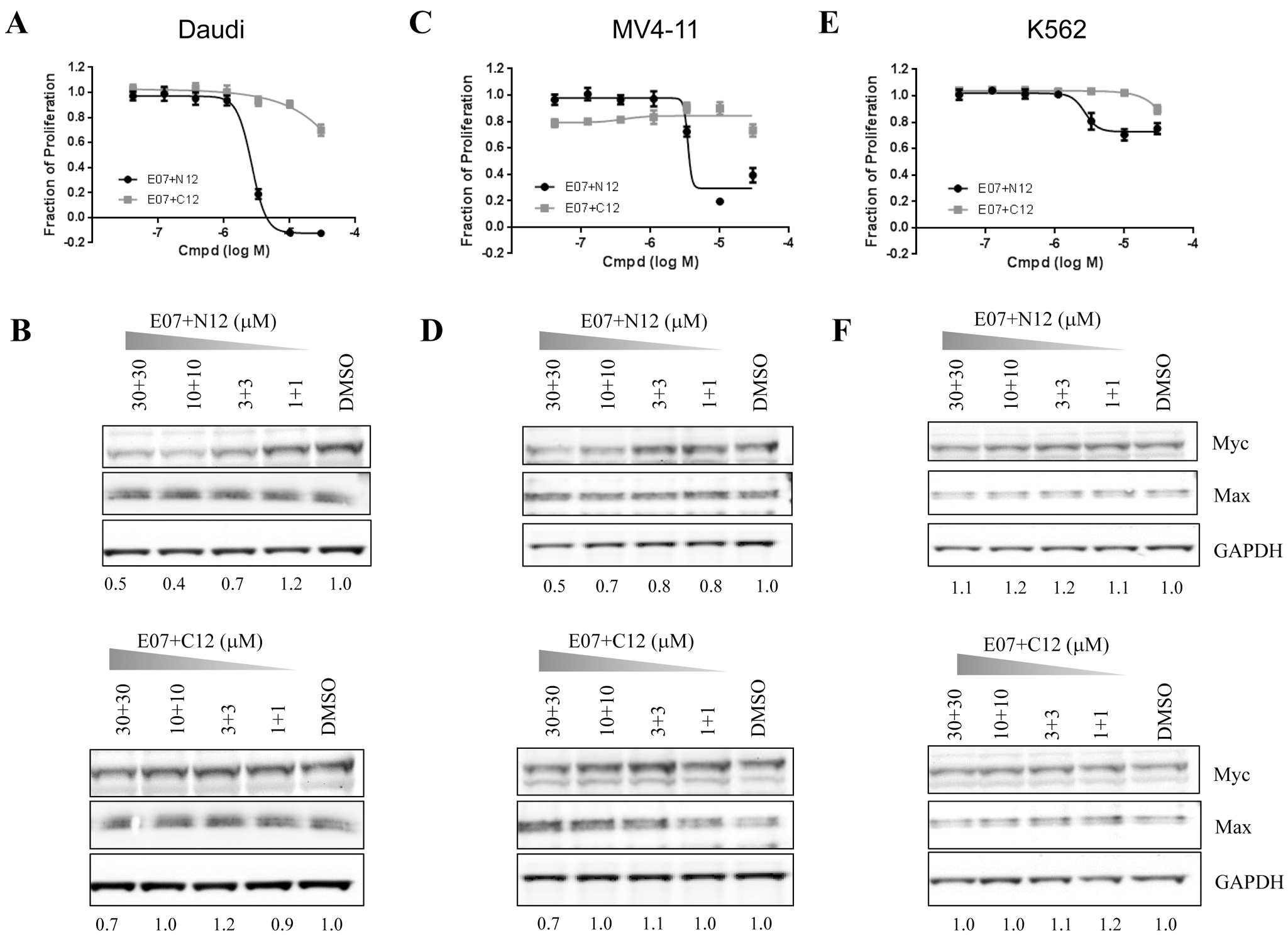

Supplement: S5 Fig — (A, C, and E) Daudi (A), MV4-11 (C)) or K562 (E) cells were treated with increasing doses of either E07+N12 or E07+C12 in a 1:1 ratio and the effect on proliferation assayed. The X-axis refers to the concentration of each individual compound, so the total inhibitor concentration will be 2 fold higher at each data point. The data is plotted as a mean ± SEM from 3 independent experiments. (B, D, and F) Daudi (B), MV4-11 (D) or K562 (F) were treated with increasing doses of either E07+N12 or E07+C12 in a 1:1 ratio and the levels of Myc, Max and GAPDH protein analyzed by western blotting after 4 hours of treatment. The relative levels of the Myc protein after correction to GAPDH levels are shown below each Western blot panel. (TIF) [file pone.0121793.s006.tif]

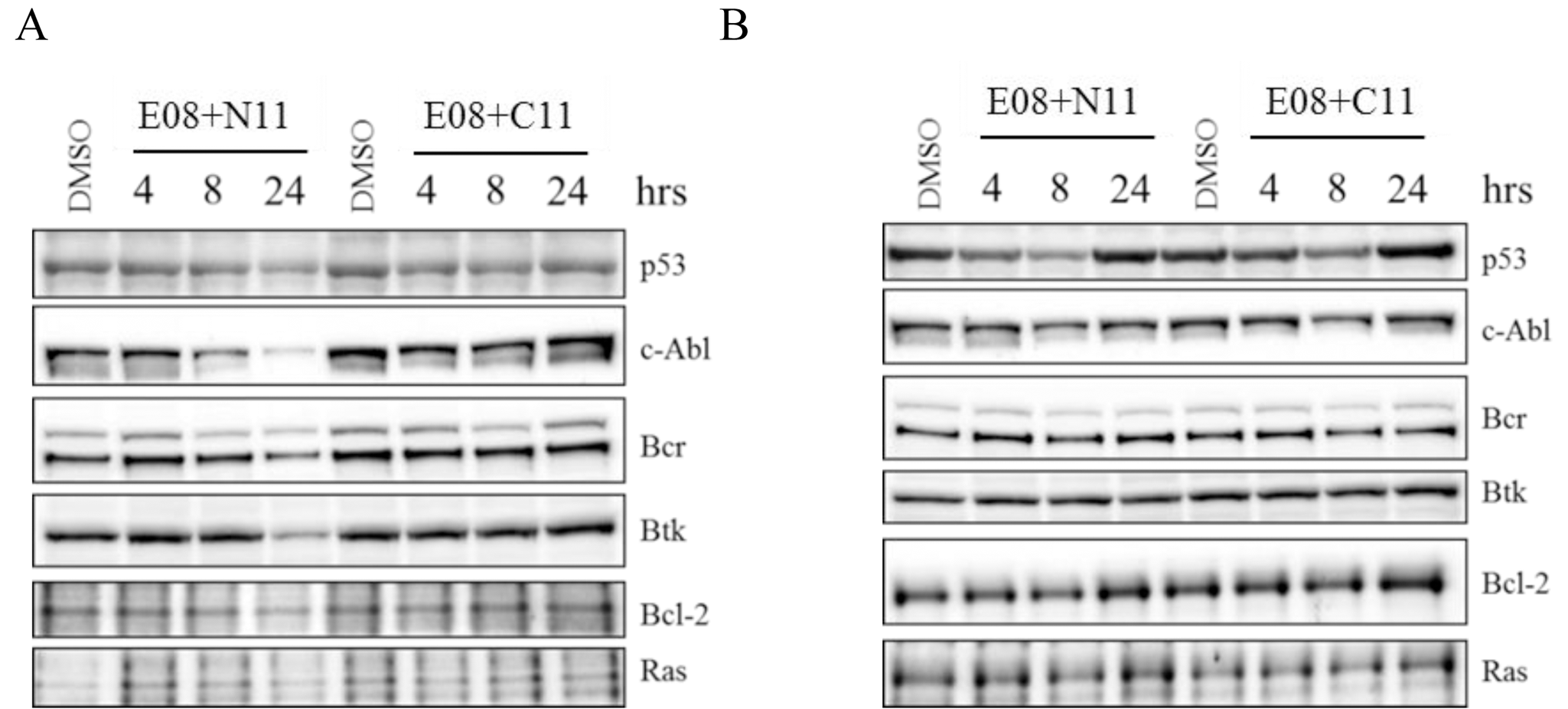

Supplement: S6 Fig — Protein lysates from the experiment shown in Fig. 5 were probed with the indicated antibodies. Daudi cells (A) and Raji cells (B) are shown. (TIF) [file pone.0121793.s007.tif]

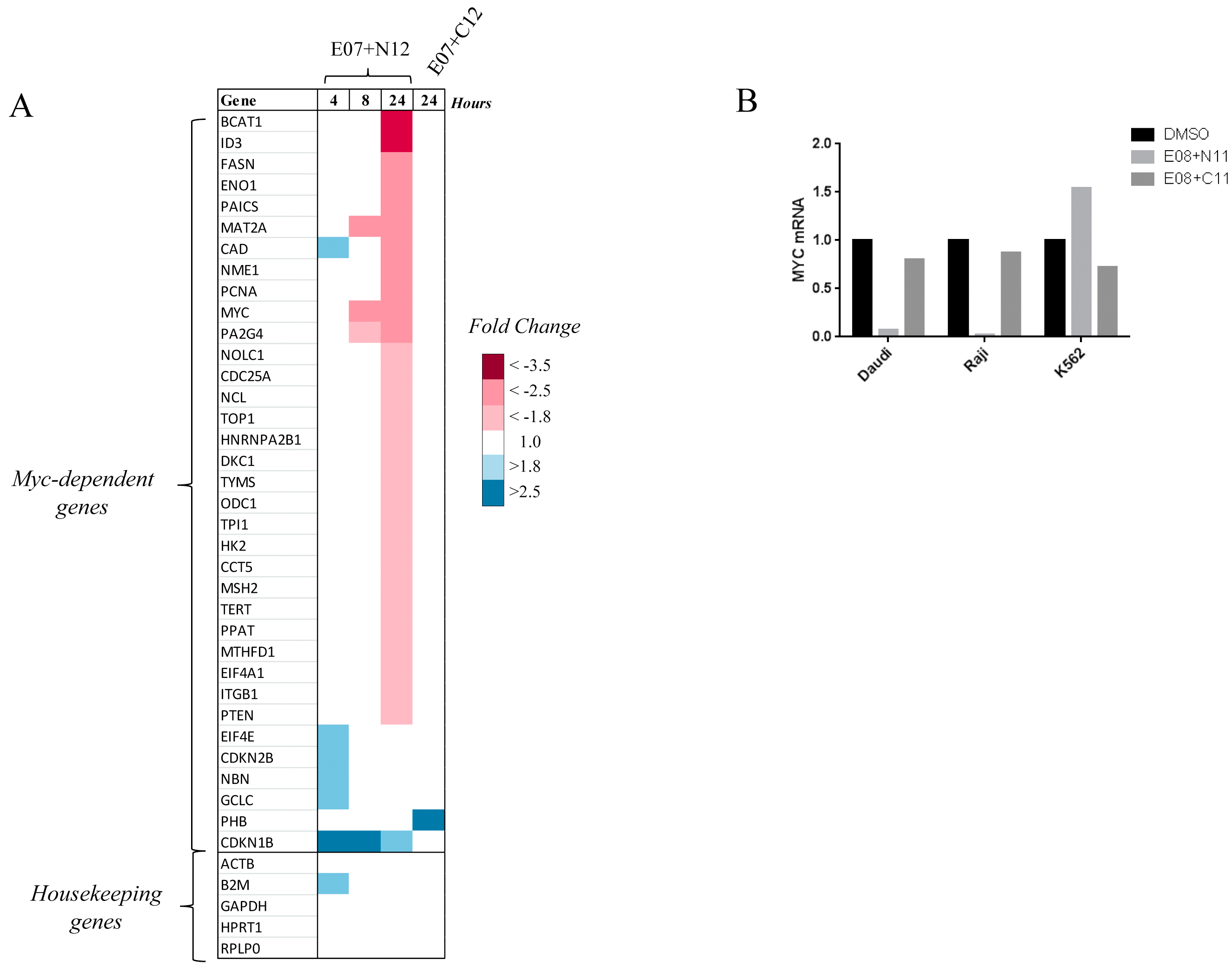

Supplement: S7 Fig — (A) Daudi cells were treated E07+N12 (10μM + 10 μM) for 4, 8 or 24 hours and the non-dimerizable control combination E07+C12 (10 μM + 10μM) for 24 hours. Gene expression levels were analyzed using a human Myc-target PCR array. Data are representative form two independent experiments. Only those genes that showed expression level changes <-1.8 or >1.8 fold, with respect to DMSO controls, at any time point or with any treatment are shown. (B) Daudi, Raji and K562 cells were treated with E08+N11 or E08+C11 (10 + 30 μM) for 24 hours and the levels of Myc mRNA analyzed by RT-PCR. (TIF) [file pone.0121793.s008.tif]
