# Supplementary material for: Reversible Linkage of Two Distinct Small Molecule Inhibitors of Myc Generates a Dimeric Inhibitor with Improved Potency That Is Active in Myc Over-Expressing Cancer Cell Lines
Source: PLoS One. 2015 Apr 15;10(4):e0121793. doi: 10.1371/journal.pone.0121793 (PMC4398458; doi:10.1371/journal.pone.0121793)
Supplement: S1 Table — (DOCX) [file pone.0121793.s009.docx]

Table S1

Parent ligand inhibition of cell-free MYC-MAX heterodimer formation^*^.

| C01 |  | >30 | | |  |
| --- | --- | --- | --- | --- | --- |
| C02 |  | >30 | | |  |
| Combination^†^ |  | 23 | ± | 8.8 | |
|  |  |  |  |  | |

^*^Average IC_50_ values (µM) with standard deviation from the MYC-MAX ELISA, as described in Experimental Procedures. ^†^Combination is an equimolar titration of C01 and C02.
